# Identification of Dhx15 as a Major Regulator of Liver Development, Regeneration, and Tumor Growth in Zebrafish and Mice

**DOI:** 10.3390/ijms25073716

**Published:** 2024-03-27

**Authors:** Irene Portolés, Jordi Ribera, Esther Fernandez-Galán, Elena Lecue, Gregori Casals, Pedro Melgar-Lesmes, Guillermo Fernández-Varo, Loreto Boix, Marco Sanduzzi, Veenu Aishwarya, Maria Reig, Wladimiro Jiménez, Manuel Morales-Ruiz

**Affiliations:** 1 Biochemistry and Molecular Genetics Department-CDB, Hospital Clínic of Barcelona, Fundació de Recerca Clínic Barcelona-Institut d’Investigacions Biomèdiques August Pi i Sunyer (FRCB-IDIBAPS), 170 Villarroel St. Barcelona, 08036 Barcelona, Spain; irene.portoles.sta@gmail.com (I.P.); jordi.ribera@ciberehd.org (J.R.); esfernandez@clinic.cat (E.F.-G.); elecuecostas@gmail.com (E.L.); casals@clinic.cat (G.C.); pmelgar@ub.edu (P.M.-L.); gfvaro@recerca.clinic.cat (G.F.-V.); wjimenez@clinic.cat (W.J.); 2 Centro de Investigación Biomédica en Red de Enfermedades Hepáticas y Digestivas (CIBERehd), 28222 Madrid, Spain; lboix@recerca.clinic.cat (L.B.); msanduzzi@clinic.cat (M.S.); mreig1@clinic.cat (M.R.); 3 Commission for the Biochemical Evaluation of the Hepatic Disease-SEQCML, 08036 Barcelona, Spain; 4 Biomedicine Department, Faculty of Medicine and Health Sciences, University of Barcelona, 08036 Barcelona, Spain; 5 Barcelona Clinic Liver Cancer Group, Liver Unit, Hospital Clinic, University of Barcelona, Institut d’Investigacions Biomèdiques August Pi i Sunyer (IDIBAPS), 08036 Barcelona, Spain; 6 AUM LifeTech, Inc., 3675 Market Street, Suite 200, Philadelphia, PA 19104, USA; veenu.aishwarya@aumbiotech.com

**Keywords:** RNA helicase, hepatocellular carcinoma, liver regeneration, liver organogenesis, glucose metabolism

## Abstract

RNA helicase DHX15 plays a significant role in vasculature development and lung metastasis in vertebrates. In addition, several studies have demonstrated the overexpression of DHX15 in the context of hepatocellular carcinoma. Therefore, we hypothesized that this helicase may play a significant role in liver regeneration, physiology, and pathology. *Dhx15* gene deficiency was generated by CRISPR/Cas9 in zebrafish and by TALEN-RNA in mice. AUM Antisense-Oligonucleotides were used to silence *Dhx15* in wild-type mice. The hepatocellular carcinoma tumor induction model was generated by subcutaneous injection of Hepa 1-6 cells. Homozygous *Dhx15* gene deficiency was lethal in zebrafish and mouse embryos. *Dhx15* gene deficiency impaired liver organogenesis in zebrafish embryos and liver regeneration after partial hepatectomy in mice. Also, heterozygous mice presented decreased number and size of liver metastasis after Hepa 1-6 cells injection compared to wild-type mice. *Dhx15* gene silencing with AUM Antisense-Oligonucleotides in wild-type mice resulted in 80% reduced expression in the liver and a significant reduction in other major organs. In addition, *Dhx15* gene silencing significantly hindered primary tumor growth in the hepatocellular carcinoma experimental model. Regarding the potential use of DHX15 as a diagnostic marker for liver disease, patients with hepatocellular carcinoma showed increased levels of DHX15 in blood samples compared with subjects without hepatic affectation. In conclusion, Dhx15 is a key regulator of liver physiology and organogenesis, is increased in the blood of cirrhotic and hepatocellular carcinoma patients, and plays a key role in controlling hepatocellular carcinoma tumor growth and expansion in experimental models.

## 1. Introduction

RNA helicases, mainly encompassing DEAD- and DEAH-box families, are highly conserved enzymes that participate in all processes of RNA metabolism, from transcription to decay, in an ATP-dependent manner. Each RNA helicase displays a specific function in a diverse number of RNA targets; however, many DEAD/DEAH-box helicases lack target specificity per se. For instance, G-patch proteins act as DEAH-box activators by binding and recruiting them to their action sites [1,2,3,4]. Upon target recognition, they exert its ATPase activity to remodel RNA [5].

We previously described that Dhx15 is a downstream target of Akt and that there is a regulatory crosstalk in the expression of both proteins [6,7]. DHX15 expression is not organ-specific and has an ubiquitous variable gene expression. DHX15 participates mainly in mRNA splicing by contributing to the dissociation of the spliceosome subunit U2 upon splicing completion. It is also known to participate in ribosome biogenesis by enhancing small subunit maturation and in viral infections by sensing double RNA strands and stimulating type I IFN and proinflammatory cytokines production [8,9].

Emerging interest has arisen in studying the interplay between helicases and cancer [10]. Mutations in splicing factors typically occur in many cancers; therefore, recent DHX15 studies have focused on elucidating its role in different types of cancer [11]. DHX15 acts as a cancer promoter in breast cancer, prostate cancer, acute myeloid leukemia, and hepatocellular carcinoma (HCC), and as an antitumor factor in glioma due to its growth inhibitory function [12,13,14,15,16]. We also described a relevant function of Dhx15 in lymphatic and blood vasculature development and functioning in vertebrates. In this context, we demonstrated the role of Dhx15 as a regulator of lung metastasis in a syngeneic mouse model of metastasis [7]. *Dhx15* gene deficiency resulted in significantly reduced metastasis due to lymphatic vascular defects and impaired endothelial energy metabolism. Further studies analyzing the specificity of DHX15 in cancer and metastasis are necessary, especially as oncologic complications are highly related to metastasis appearance.

Despite the increasing interest in DHX15 and the role played by its upstream regulator Akt in liver function, regeneration, and cytoprotection [17,18,19], this helicase had not been studied in the liver. Recently, two different studies evaluated DHX15 expression in hepatocellular carcinoma patients, showing a differential expression of this helicase. Xie C. et al. described significant overexpression of DHX15 in human primary HCC correlated with poor survival [16]. Later, Zhao M. et al. described DHX15 as an inhibitor of autophagy that was less expressed in HCC tumor tissues [20]. Although both studies show contrasting results, these observations suggest that DHX15 is a protein target for HCC. Thus, it would be necessary to decipher the function of DHX15 in the liver and specifically in the context of HCC. Here, we explore its role in the liver in two different animal models, zebrafish and mouse. We analyze the effects of *Dhx15* knockdown in liver organogenesis, liver vasculature, and liver regeneration. Furthermore, we evaluate the functions of Dhx15 in HCC and liver metastasis, in *Dhx15* heterozygous mice and using the self-delivering AUM*silence* ASO technology, providing evidence for its role in the regulation of metastasis and primary tumor growth.

## 2. Results

### 2.1. Impaired Liver Development in Dhx15-Deficient Zebrafish Embryos

**Figure 1:**
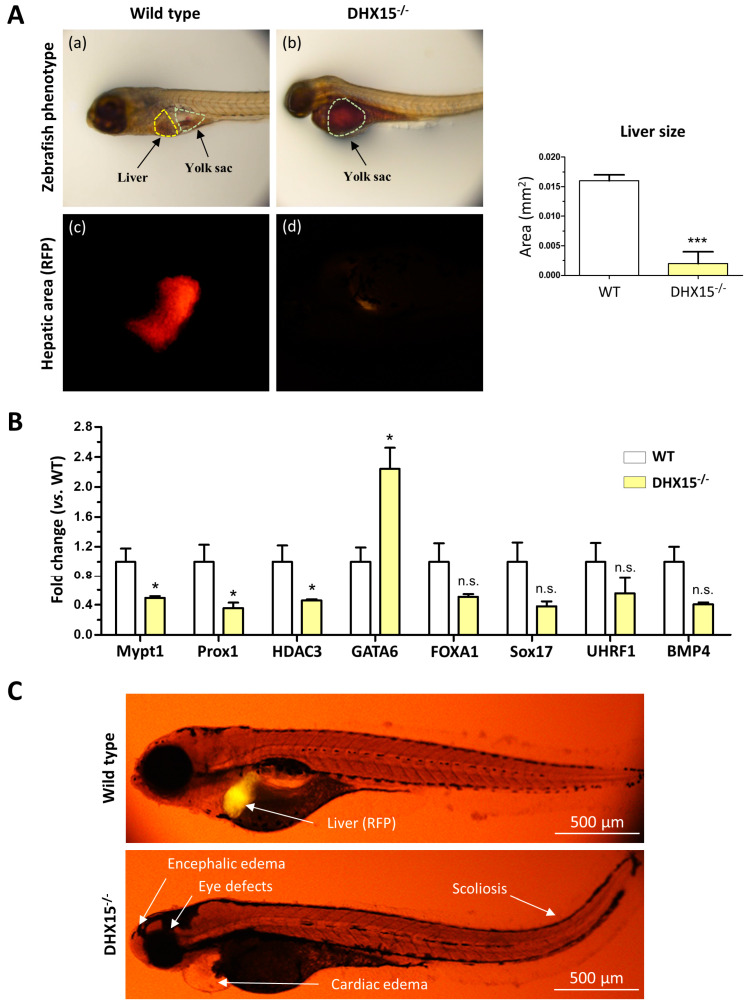
*Dhx15* gene deficiency in zebrafish resulted in a liverless phenotype. (**A**) In the upper panels, representative images of wild-type (panel a) and *Dhx15*^−/−^ (panel b) larvae at 5 day post fertilization (dpf) revealing an absence of liver development and metabolite retention in the yolk sac. Each area is enclosed with different colors (yellow lines correspond to liver region and green lines to yolk sac). In the lower panels, positive liver red fluorescence in wild-type (panel c) and *Dhx15*^−/−^ (panel d) larvae. Quantification of liver size is shown in adjacent graph. Bars represent the mean ± SEM, *** *p* < 0.001 vs. wild-type zebrafish (n = 15). (**B**) RNA extraction of zebrafish embryos at 4 dpf from either wild-type or *Dhx15* knockout larvae was performed. mRNA expression was analyzed by RT-qPCR. The graph shows the expression levels of the *Mypt1*, *Prox1*, *Hdac3*, *Gata6*, *Foxa1*, *Sox17*, *Uhrf1,* and *Bmp4* genes in the *Dhx15*^+/+^ and *Dhx15*^−/−^ conditions. mRNA levels are shown as fold change relative to *Actin* mRNA levels. Bars represent the mean ± SEM, * *p* < 0.05 vs. wild-type(n = 4). N.S. not significant. (**C**) Representative images comparing wild-type and *Dhx15*^−/−^ larvae at 7 dpf; *Dhx15*^−/−^ larvae show absence of liver (red fluorescence) and morphological defects including encephalic and cardiac edema, scoliosis, and impaired neural/eye growth.

As we and others previously described, *Dhx15* deficiency in zebrafish embryos depicts phenotypic abnormalities and ultimately results in lethality at day 8 post-fertilization (dpf). Such mutant embryos are characterized by encephalic and cardiac edema, scoliosis, impaired neural/eye growth. and defective pectoral fin and jaw development [7,21]. To determine whether *Dhx15* gene deficiency is also associated with liver organogenesis, we generated a *Dhx15* knockout zebrafish model in a red fluorescent protein (RFP) background under the liver-specific promoter fab10. At 5 dpf, *Dhx15*^−/−^ embryos presented evident differences from their wild-type (WT) clutch-mates; at this stage, mutant embryos lack livers (Figure 1A). By means of fluorescent microscopy, we specifically visualized the hepatic area marked with RFP in zebrafish embryos, and we detected residual or no staining tissue in *Dhx15*^−/−^ embryos (Figure 1A, panels c and d). We also determined the percentage of hepatic yolk retention. *Dhx15*^−/−^ embryos depicted a 100% yolk retention; in contrast, WT clutch-mates presented a 0–5% yolk retention. This observation suggests that the lack of liver tissue in *Dhx15*^−/−^ embryos may be the mechanism responsible for yolk retention due to impaired nutrient metabolism, causing metabolite accumulation in the yolk sac (Figure 1A, panel b). Heterozygous embryos were also evaluated and displayed similar characteristics as WT embryos, indicating no evident alterations in heterozygosis (Supplementary Figure S1).

To evaluate whether the absence of liver in *Dhx15*^−/−^ embryos was caused by genetic alterations induced by *Dhx15*-deficiency, we evaluated the gene expression of key mediators of liver development, such as, *Prox1*, *Mypt1*, *Hdac3*, *Foxa1*, *Sox17*, *Uhrf1,* and *Bmp4*. We found that *Mypt1*, *Prox1,* and *Hdac3* were significantly downregulated in *Dhx15*^−/−^ embryos, while only *Gata6* was significantly upregulated compared to WT embryos. Other genes depicted a nonsignificant tendency towards a reduced expression (Figure 1B).

In Figure 1C, the morphological differences between WT and mutant embryos and the absence of liver in *Dhx15*^−/−^ larvae are depicted. Such results urged us to elucidate the role of Dhx15 in the liver.

### 2.2. Altered Liver Vasculature in Dhx15-Partially-Deficient Mice

**Figure 2:**
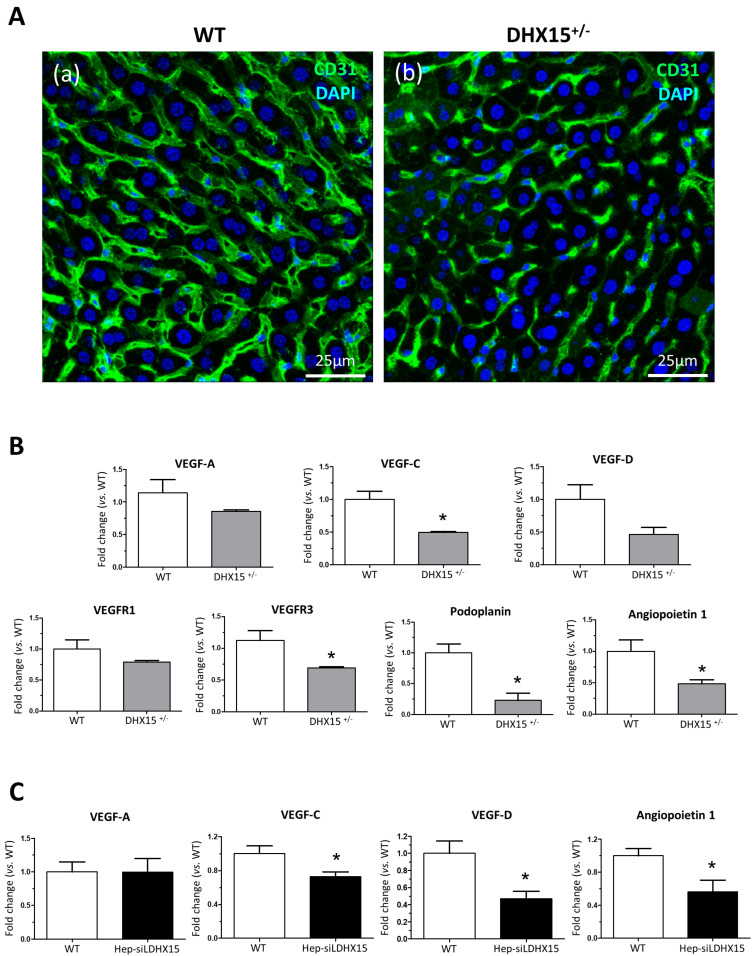
Intrahepatic liver vasculature was altered in *Dhx15*^+/−^ mice. (**A**) Representative Cd31 liver immunostaining (green) for wild-type (panel a) and *Dhx15*^+/−^ (panel b) mouse. Nuclei counterstaining was performed with DAPI (blue). Confocal microscope, original magnification: 300×. (**B**) RNA extraction of liver tissue from either wild-type or *Dhx15*^+/−^ mice was performed. mRNA expression was analyzed by RT-qPCR. The graphs show the expression levels of *Vegf-a*, *Vegf-c*, *Vegf-d*, *Vegfr1*, *Vegfr3*, *Podoplanin,* and *Angiopoietin 1* genes in the wild-type and *Dhx15*^+/−^ conditions. mRNA levels are shown as fold change relative to *Hprt* mRNA levels. Bars represent mean ± SEM, * *p* < 0.05 vs. wild-type (n = 4). (**C**) RNA extraction of the hepatocyte cell line without or with silenced *Dhx15* gene was performed. mRNA expression was analyzed by RT-qPCR. The graphs show the expression levels of *Vegf-a*, *Vegf-c*, *Vegf-d,* and *Angiopoietin 1* genes in wild-type and *Dhx15* silenced conditions. mRNA levels are shown as fold change relative to *Hprt* mRNA levels. Bars represent mean ± SEM, * *p* < 0.05 vs. wild-type (n = 4).

In a prior study, we described Dhx15-related vascular defects in mutant zebrafish that were also occurring during development [7]. In the present study, we wanted to further expand these previous results by studying the vasculature morphology within the liver. Since our *Dhx15*^−/−^ zebrafish model presented defects in liver organogenesis, we investigated the impact of the partial Dhx15 deficiency on liver function and hepatic angioarchitecture in adult *Dhx15*^+/−^ mice, which are viable, compared with *Dhx15*^−/−^ mice that showed embryonic lethality [7]. Supplementary Figure S2 includes gross liver images, body weight, liver weight, and graphs depicting concentrations of albumin, total bilirubin, and blood urea nitrogen, which are classic parameters for evaluating liver function. Our results indicate that heterozygous Dhx15 deficiency does not negatively impact organ function, as evidenced by the absence of significant changes in these parameters compared with wild-type mice. Regarding the evaluation of hepatic angioarchitecture, we observed differences in the thickness and expansion of the liver vasculature in *Dhx15*^+/−^ mice, compared to WT (Figure 2A), consisting of the presence of thinner vessels and lower vascular connectivity.

To study the molecular variations associated with the intrahepatic vascular alterations, we quantified RNA expression of different key factors involved in the generation and maintenance of blood and lymphatic vessels. In heterozygous mice, we observed a reduced RNA expression of *Vegf-c*, *Vegfr3*, *Podoplanin,* and *Angiopoietin 1* and a nonsignificant tendency towards a lower expression of *Vegf-d* and *Prox1* (Figure 2B). Similarly to the results found in hepatic tissue, we detected a reduced expression of *Vegf-c*, *Vegf-d,* and *Angiopoietin 1* in a hepatocyte cell line with the *Dhx15* gene silenced (Hep-siDhx15), compared to WT hepatocytes (Figure 2C).

### 2.3. Dhx15 Partial Gene Deficiency Decreases the Regenerative Capacity of the Liver in Mice

**Figure 3:**
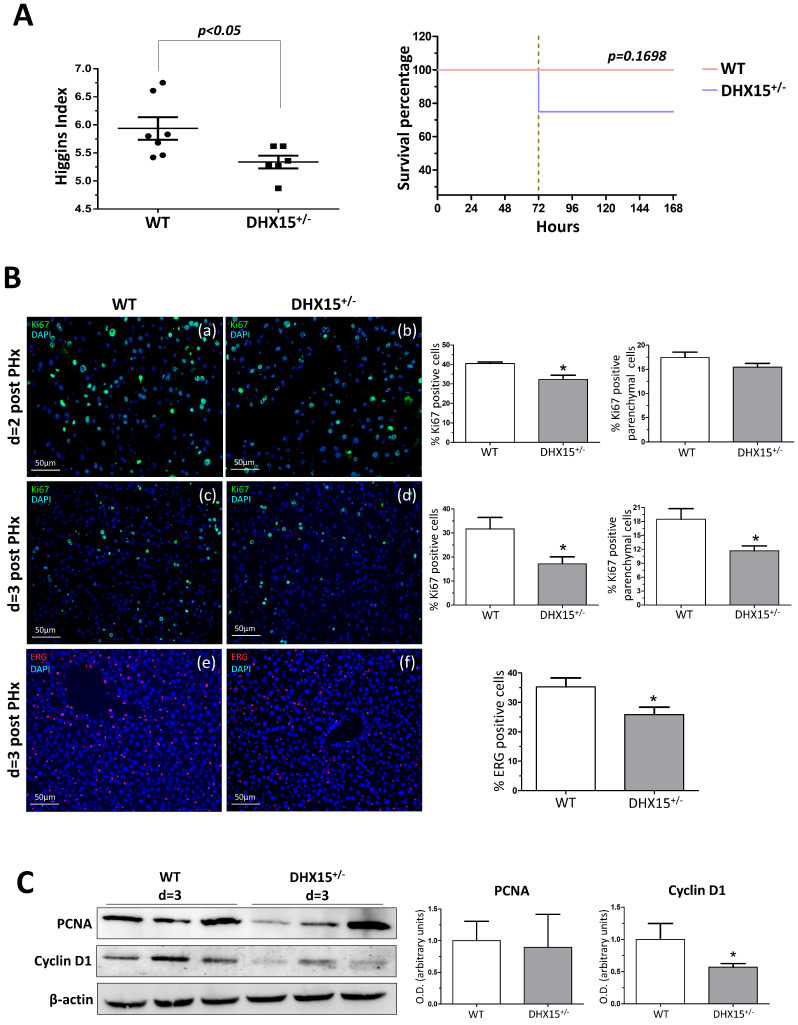
*Dhx15* genetic deficiency impaired liver regeneration in mice after PHx. (**A**) On the left graph, hepatic regenerative index (liver weight/total body weight) obtained in WT and *Dhx15*^+/−^ mice 7 days after PHx. On the right graph, survival curves from WT and *Dhx15*^+/−^ mice after PHx generated using the product limit method of Kaplan and Meier. Survival curves were compared using the log-rank test. (**B**) In the upper panels, representative merged images of immunofluorescence staining of Ki67-positive cells (green) and DAPI (blue) in WT and *Dhx15*^+/−^ livers at 2 (panels a and b) and 3 (panels c and d) days following PHx. In the lower panels (e and f) are representative merged images of immunofluorescence staining of Erg-positive cells (red) and DAPI (blue) in WT and *Dhx15*^+/−^ livers at 3 days following PHx. Original magnification ×200. The graph shows the computer-assisted quantification of Ki67 and Erg-positive cells/total nuclei at different times following PHx. We differentiated hepatocytes from the rest of the nonparenchymal cells stained positively for Ki67 by the exclusion of smaller nuclear size with the ImageJ software (version 1.53t). Bars represent mean ± SEM, * *p* < 0.05 vs. wild-type (n = 4). (**C**) Expression of Pcna and Cyclin d1 proteins was evaluated by Western blot using liver tissue lysates from wild-type and *Dhx15*^+/−^ mice 3 days after PHx. β-actin was used as a loading control. Densitometric analysis of protein expression is shown on the bar graph. Bars represent mean ± SEM, * *p* < 0.05 vs. wild-type (n = 3).

To study the role of Dhx15 during regeneration, we performed two-thirds partial hepatectomy (PHx) in WT and *Dhx15*^+/−^ mice. Mice were sacrificed at 2, 3, and 7 days post-PHx; the wet remnant liver weight together with the total body weight was used to calculate the hepatic regenerative rate known as Higgins Index. Seven days after PHx, *Dhx15*^+/−^ mice showed decreased regenerative rate associated with a trend of increasing mortality at 72 h after surgery compared with WT mice (Figure 3A), although these differences were not significant.

Liver tissue samples obtained at day 2 and 3 after PHx were used to evaluate cell proliferation by Ki67 immunostaining. In *Dhx15*^+/−^ mice at day 2 post-PHx, we observed a slight but significant decrease in total cell proliferation compared to WT mice (Figure 3B, panels a and b), although this tendency was not significant for parenchymal cells. At day 3 post-PHx, we observed evident and significant reduction in proliferation in both parenchymal and total cells in *Dhx15*^+/−^ mice compared with WT mice (Figure 3B, panels c and d). Next, we specifically evaluated the presence of endothelial cells in the regenerating liver at 3 days post-PHx by ETS related gene (Erg) immunostaining. We observed a significant reduction in Erg-positive cells in the heterozygous livers compared to WT (Figure 3B, panels e and f). The reduced proliferation was accordingly associated with a reduced protein expression of Cyclin d1 but not Pcna (Figure 3C).

### 2.4. Dhx15 Deficiency Alters Glucose Metabolism

Previous RNAseq and proteomic analysis results in *Dhx15* silenced endothelial cells led us to conclude that Dhx15 participates in carbohydrate metabolism (Ribera J. 2021 [7]) (Supplementary Figure S3). To further validate these previous published results, we performed functional metabolic analyses in *Dhx15*^+/−^ mice.

**Figure 4:**
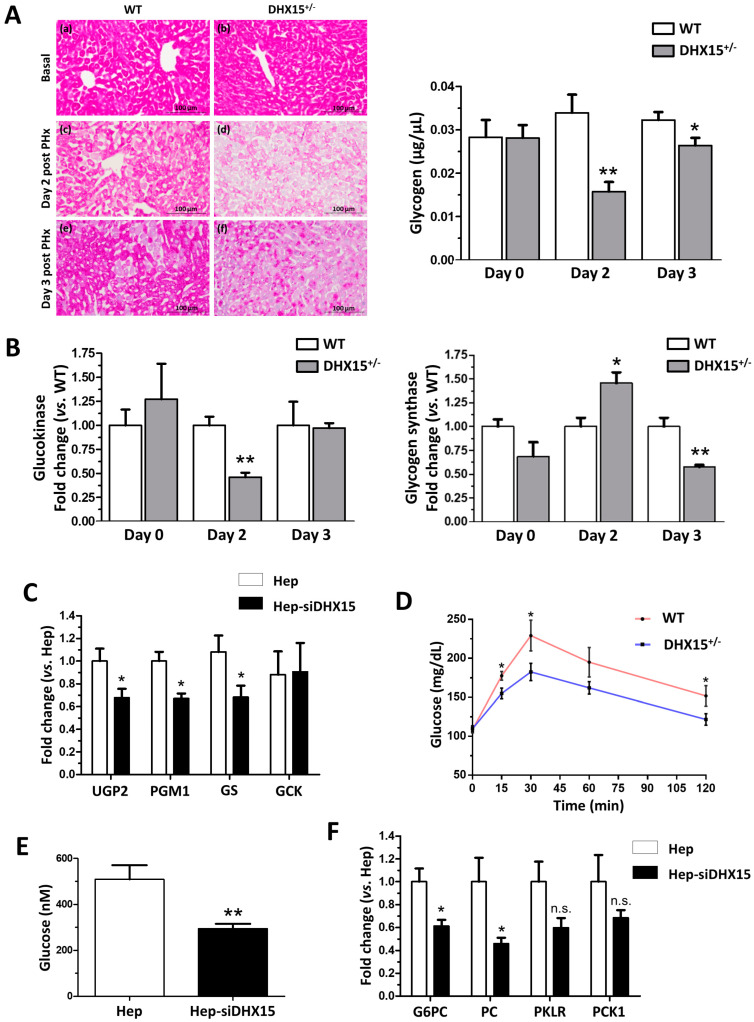
Impaired glucose metabolism in *Dhx15*^+/−^ mice. (**A**) On the left, images from periodic acid–Schiff (PAS) staining of wild-type and *Dhx15*^+/−^ mice in basal condition (upper panels), 2 days after PHx (middle panels) and 3 days after PHx (lower panels). On the right, glycogen in the hepatic tissue of wild-type and *Dhx15*^+/−^ mice at 0, 2, and 3 days after PHx measured by colorimetric assay. Bars represent mean ± SEM, * *p* < 0.05 and ** *p* < 0.01 vs. wild-type at the same time points (n = 6). (**B**) RNA extraction of liver tissue from either wild-type or *Dhx15*^+/−^ mice was performed before and 2 and 3 days after PHx. mRNA expression was analyzed by RT-qPCR. The graph shows the expression levels of glucokinase and glycogen synthase genes in the wild-type and *Dhx15*^+/−^ mice. mRNA levels are shown as fold change relative to *Hprt* mRNA levels. Bars represent mean ± SEM, * *p* < 0.05 and ** *p* < 0.01 vs. wild-type at the same time points (n = 6). (**C**) RNA extraction of the hepatocyte cell line without or with silenced *Dhx15* gene was performed. mRNA expression was analyzed by RT-qPCR. The graph shows the expression levels of *Ugp2*, *Pgm1*, *Gs,* and *Gck* genes in wild-type and *Dhx15* silenced conditions. mRNA levels are shown as fold change relative to *Hprt* mRNA levels. Bars represent mean ± SEM, * *p* < 0.05 vs. wild-type (n = 6). (**D**) Pyruvate tolerance test performed in wild-type and *Dhx15*^+/−^ mice with an intraperitoneal injection of sodium pyruvate (2.0 g/kg body weight in 1 x PBS) after overnight fasting. Blood glucose levels were measured at 0, 15, 30, 60, and 120 min. Bars represent mean ± SEM, * *p* < 0.05 vs. wild-type (n = 15). (**E**) Levels of intracellular glucose production in the hepatocyte cell line without or with silenced *Dhx15* gene measured by colorimetric assay. Bars represent mean ± SEM, ** *p* < 0.01 vs. wild-type (n = 3). (**F**) RNA extraction of the hepatocyte cell line without or with the *Dhx15* gene silenced was performed. mRNA expression was analyzed by RT-qPCR. The graph shows the expression levels of *G6pc*, *Pc*, *Pklr,* and *Pck1* genes in the wild-type and *Dhx15* silenced conditions. mRNA levels are shown as fold change relative to *Hprt* mRNA levels. Bars represent the mean ± SEM, * *p* < 0.05 vs. wild-type (n = 4). N.S. not significant.

After partial hepatectomy, glycogen storage is used to feed the highly metabolic demand of liver regeneration, finding its lowest peak at 24 h post-PHx, to later recover its normal levels [22,23]. To elucidate whether the defects in regeneration were related to metabolic alterations restricting the energetic demands in the liver, we first quantified glycogen levels in the regenerating livers of our *Dhx15*-deficient animal experimental model. We observed a significant decrease in glycogen in *Dhx15*^+/−^ mice two and three days after PHx compared to WT (Figure 4A), indicating that heterozygous mice are unable to normalize glycogen levels post-PHx. We analyzed the expression of glucokinase (*Gck*), which converts glucose into glucose 6-phosphate, and the expression of glycogen synthase (*Gs*) which converts UDP-glucose into glycogen, before and after hepatectomy (Figure 4B). We observed that after hepatectomy, *Dhx15*^+/−^ mice showed significantly lower levels of *Gck* at day 2, returning to basal levels at day 3. On the other hand, the *Gs* gene showed differential expression in the context of *Dhx15* deficiency with overexpression on day two and gene repression on day three, compared to the wild-type group. Although *Gs* does not exhibit a defined tendency of differential expression, its consistent transcriptional deregulation together with the differential expression of *Gck* are concordant with the differences found in glycogen accumulation between the wild-type and the *Dhx15*^+/−^ groups at different time points (Figure 4A). This may indicate that the deficiency of the Dhx15 helicase is altering the expression of *Gck* and that *Gs* expression increases in order to compensate such enzymatic deficiency; however, it is not able to properly restore the glycogen storage levels. Accordingly, in *Dhx15* silenced hepatocytes (Hep-siDhx15), we evaluated genes participating in glycogenesis and found lower RNA expression of *Phosphoglucomutase 1* and *Udp-glucose pyrophosphorylase* that participate in the conversion of glucose into glycogen (Figure 4C).

In a previous Dhx15 study we observed decreased mitochondrial activity and ATP production in endothelial cells [7]. We evaluated now if glucose metabolism might be affected by Dhx15 depletion, possibly reducing hepatocyte proliferation during regeneration. *Dhx15*^+/−^ mice presented a lower glucose production after pyruvate injection compared to WT mice, reaching lower levels of maximal glucose production (Figure 4D). We quantified glucose production in Hep-siDhx15 and observed a significant decreased glucose production caused by Dhx15 deficiency (Figure 4E). To confirm these results, we evaluated the expression of glycolysis-participating enzymes in Hep-siDhx15; we found decreased expression of *Glucose-6-phosphatase* (*G6pc*) and *Pyruvate carboxylase* (*Pc*) which participate in the gluconeogenic conversion of pyruvate into glucose (Figure 4F), and we detected no differences in *Pyruvate kinase* (*Pklr*) and *Phosphoenolpyruvate carboxykinase* (*Pck*). These results evidence the metabolic alterations caused by Dhx15 partial deficiency.

### 2.5. Dhx15-Related Vascular Alterations Derive in Less Hepatic Tumor Nodule Events in an HCC Mouse Model

**Figure 5:**
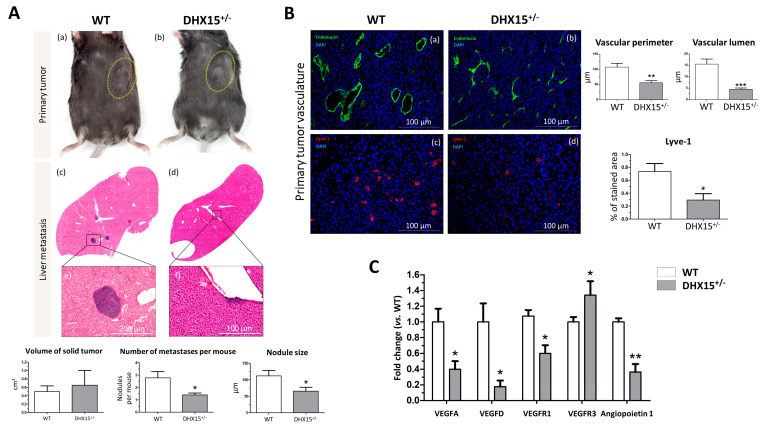
Tumor growth and metastases in *Dhx15*^+/−^ mice following Hepa1-6 tumor induction. (**A**) In the upper panels, macroscopic images of tumor size in wild-type (panel a) and *Dhx15*^+/−^ mice (panel b) 5 weeks after mouse Hepa1-6 hepatoma cells implantation. Yellow circles delimitate primary tumor localization. On the lower panels, representative liver sections with metastatic areas after haematoxylin–eosin staining (H&E) in wild-type (panel c) and *Dhx15*^+/−^ mice (panel d). Original magnification: ×10. The lower images of each condition correspond to the enclosed area of the upper images that were taken at higher magnifications (×100 and ×200, respectively). Quantifications of tumor volume (cm^3^), number of metastases, and nodule size are shown in the lower graphs. Bars represent mean ± SEM, * *p* < 0.05 vs. wild-type mice (n = 10 animals for each condition). (**B**) Immunostaining of intratumoral vessels in wild-type (blood vessels stained with endomucin, panel a, and lymphatic vessels stained with Lyve-1, panel c) and *Dhx15*^+/−^ (blood vessels stained with endomucin, panel b, and lymphatic vessels stained with Lyve-1, panel d) mice. Quantification of total vascular perimeter and lumen of all intratumoral blood vessels and percentage of Lyve-1-positive immunostaining are shown in the right graphs. Bars represent mean ± SEM, * *p* < 0.05, ** *p* < 0.01, *** *p* < 0.001 vs. wild-type mice (n = 10 animals for each condition). Original magnification: 200×. (**C**) RNA extraction of primary tumors from either wild-type or *Dhx15*^+/−^ mice was performed. mRNA expression was analyzed by RT-qPCR. The graph shows the expression levels of *Vegf-a*, *Vegf-d*, *Vegfr1*, *Vegfr3,* and *Angiopoietin 1* genes in wild-type and *Dhx15*^+/−^ conditions. mRNA levels are shown as fold change relative to *Hprt* mRNA levels. Bars represent mean ± SEM, * *p* < 0.05, ** *p* < 0.01 vs. wild-type (n = 4).

Recent studies evaluate the role of DHX15 in different cancer types. In hepatocellular carcinoma (HCC), DHX15 was found to be overexpressed in human cancerous livers [16]. Also, Zhao et al. described that DHX15 in HCC has an inhibitor role in HCC proliferation by suppressing autophagy in a hepatoma cell line [20]. Since we previously found lymphatic alterations and reduced metastasis in a lung cancer mouse model in *Dhx15* heterozygous mice [7], we now evaluated the role of Dhx15 in HCC and liver metastasis in mice. We benefited from the use of the murine hepatocellular carcinoma cell line named Hepa 1-6 to establish a syngeneic cancer model. Upon subcutaneous injection of Hepa 1-6 cells in the flank of WT and *Dhx15*^+/−^ mice, we followed primary tumor formation. Five weeks after injection, *Dhx15*^+/−^ mice exhibited similar tumor size (Figure 5A, panels a and b) and low, nonsignificant expression levels of the HCC marker alpha-fetoprotein (AFP) compared to wild-type mice (Supplementary Figure S4A). We evaluated tumor invasion of the liver by Hepa 1-6 and found small nodules within the livers of *Dhx15*^+/−^ mice compared to WT mice (65.62 ± 11.77 vs. 111 ± 15.92 µm of nodule size per mouse, respectively; *p* < 0.05). The liver nodules in WT mice were larger and more numerous (Figure 5A, panels c and d). *Dhx15*^+/−^ mice presented fewer invasive events in the liver compared to WT mice (1.40 ± 0.16 vs. 2.78 ± 0.49 number of nodules per mouse, respectively; *p* < 0.05).

We also studied the lymphatic and blood vasculature within the primary tumor to determine if differences in tumor nodule invasion were related to aberrant vasculature structures. In accordance with reduced tumor nodule formation, we observed clear differences in WT and heterozygous mice, depicting vascular abnormalities in *Dhx15*^+/−^ mice. We studied both endothelial and hematopoietic vasculature by endomucin staining (Figure 5B, panels a and b). In *Dhx15*^+/−^ tumors, we observed smaller vases with a reduced lumen. We next studied the organization of lymphatic cells by Lyve-1 immunostaining. Comparing WT and *Dhx15*^+/−^ mice, the heterozygous mice presented a significant decrease in % stained area with Lyve-1 (Figure 5B, panels c and d).

To better analyze lymphatic defects, we quantified the RNA expression of several vascular factors within the primary tumor. We analyzed the expression of *Vegf-d* which participates in lymphangiogenesis and in endothelial cell growth, being relevant in the development of new lymphatic vasculature in metastasis [24]. We found a significant decrease in *Vegf-d* and a significant upregulation of its receptor *Vegfr3* in the primary tumors of *Dhx15*^+/−^ mice. We also found a significant decreased expression of *Vegf-a*, *Vegfr1*, its receptor, and angiopoietin 1, in the primary tumors of *Dhx15*^+/−^ mice (Figure 5C). The results suggest that the fewer hepatic tumor nodules observed in *Dhx15*^+/−^ mice might be due to an impaired growth and development of the vascular network in the primary tumor.

### 2.6. AUMsilence ASO Mediated Dhx15 Silencing in Mice Reduces Primary Tumor Volume in an HCC Mouse Model

Following previous observations by us and others regarding the function of DHX15 in HCC, we decided to evaluate the potential therapeutic use of Dhx15 deletion in the cancer setting. To do so, we studied the effect of the AUM*silence* antisense oligonucleotides (AUM*silence^TM^* ASO) silencing methodology [25] to silence *Dhx15* in vivo. Upon a single dose of AUM*silence* Dhx15 intravenous injection, we obtained an 80% of Dhx15 silencing in the liver that was maintained up until 72 h post-injection (Supplementary Figure S4B). Dhx15 silencing was also evaluated in the lungs and spleen, where we observed a milder Dhx15 deletion.

**Figure 6:**
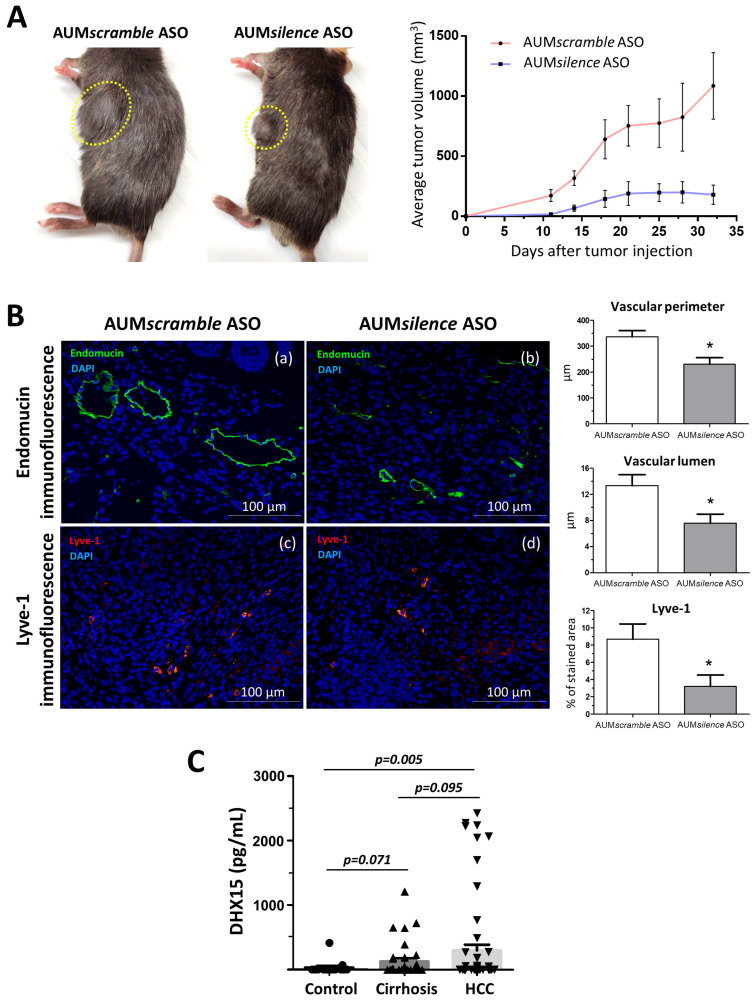
Tumor growth in wild-type mice following Hepa1-6 tumor induction after *Dhx15* inhibition. (**A**) On the left, macroscopic images of tumor size in wild-type AUMscramble ASO (scramble) and AUMsilence ASO (*Dhx15* specific)-injected mice 5 weeks after mouse Hepa1-6 hepatoma cells implantation. Yellow circles delimitate primary tumor localization. On the right, the graph shows tumor growth in wild-type AUMsilence ASO (*Dhx15*-specific) or AUM*scramble* ASO (scramble)-injected mice. Tumor volume was monitored every 3 days until the end of the study. (**B**) Immunostaining of intratumoral vessels (blood vessels stained with endomucin, panels a and b, and lymphatic vessels stained with anti-Lyve-1 antibody, panels c and d) in wild-type AUMsilence ASO (*Dhx15*-specific) or AUM*scramble*-ASO (scramble)-injected mice. Quantifications of total vascular perimeter and lumen of all intratumoral blood vessels, and percentage of Lyve-1-positive immunostaining are shown in the right graph. Bars represent mean ± SEM, * *p* < 0.05 vs. wild-type mice (n = 10 animals for each condition). Original magnification: 200×. (**C**) DHX15 levels in the serum of patients with cirrhosis (n = 35), hepatocellular carcinoma (n = 62), and healthy controls (n = 24).

To evaluate the effects of a strong Dhx15 inhibition in the mouse liver in a cancerous context, we established the Hepa 1-6 HCC model in AUM*silence* ASO-injected mice. We followed primary tumor growth and observed a significantly reduced tumor volume in the AUM*silence* Dhx15 injected mice compared to AUM*scramble* scramble group (AUM*scramble* ASO) (179.6 ± 80.06 vs. 1085 ± 277.1 mm^3^ primary tumor volume five weeks post-implantation, respectively; *p* < 0.01; Figure 6A). In agreement with the results found in *Dhx15*^+/−^ mice, we also observed a reduction in several vascular genes in the livers of AUM*silence* ASO Dhx15 injected mice, such as *Vegf-a*, *Vegf-d*, *Vegfr1*, and *Vegfr3* (Supplementary Figure S4C).

We also analyzed the vascular angioarchitecture of the primary tumor and, similarly to the HCC model in *Dhx15*^+/−^ mouse, we observed alterations in the vasculature in terms of a significantly reduced vascular perimeter and lumen of blood vessels detected by endomucin immunostaining and a significant reduction in lymphatic vessels detected by Lyve-1 immunostaining in the AUM*silence* ASO Dhx15 group compared to the AUM*scramble* ASO scramble group (Figure 6B).

Today, the biochemical diagnosis of hepatocellular carcinoma continues to be a challenge. Due to the effect of Dhx15 on tumor growth in our experimental model of HCC, we wanted to evaluate the differential diagnostic utility of this marker in patients. With this purpose, we performed serological evaluation of DHX15 levels by ELISA in a cohort of patients with different liver disease etiologies. As shown in Figure 6C, patients with HCC present significant higher levels of circulating DHX15 compared to healthy subjects (300.3 ± 88.2 vs. 32.4 ± 27.7 pg/mL; *p* < 0.01; respectively). We also detected a trend of increased circulating values of DHX15 in patients with HCC compared to cirrhotic patients without hepatic tumors, although this trend was not significant (300.3 ± 88.2 vs. 132.0 ± 46.2 pg/mL; *p* = 0.095; respectively).

## 3. Discussion

The DEAH-box RNA helicase DHX15 is implicated in diverse biological functions. In this study, we describe a total impairment of liver development caused by the mutation of *Dhx15* in our CRISPR/Cas9 zebrafish. To our knowledge, Dhx15 had not previously been described to play a role in liver development; now, we report, for the first time, a lack of the hepatic organ in zebrafish due to *Dhx15* knockout. This implies a redundant and noncompensated function of Dhx15 in liver development. Since DHX15 is a splicing factor, we studied whether *Dhx15* knockout was affecting genes classically described to be implicated in liver formation in zebrafish. We found reduced expression of *Prox1*, which is one of the earliest markers for definitive hepatoblasts*, Mypt1,* which participates in bud formation, and *Hdac3,* which participates in liver budding and differentiation [26], in *Dhx15*^−/−^ embryos at 4dpf. Knockout experiments in zebrafish have helped to determine the crucial and nonredundant function of several genes in liver development, such as *Gata 4* and *6*, *Hdac3*, *Hhex,* and *Mypt1*. Single mutation of these genes results in major hepatic development complications [26,27,28,29]. For instance, Huang H. et al. described a liverless phenotype in *Mypt1* mutant zebrafish [30]. In that study, *Mypt1* mutation caused hepatoblast apoptosis that resulted in blockage of liver bud formation. Here, we added *Dhx15* to the list of essential genes in liver development in zebrafish. Furthermore, we observed a regulatory crosstalk between Dhx15 and *Mypt1*, *Prox1,* and *Hdac3*, thus supporting the role of Dhx15 in hepatic organogenesis. More studies are needed to evaluate the contribution of each of these factors in the final effect on organogenesis.

In *Dhx15*^−/−^ zebrafish embryos, liver absence causes retention of metabolites in the yolk sac. At early embryonic stages, zebrafish energy demands are supplied by the metabolization of nutrients that takes place in the liver [31]. The *Dhx15*^−/−^ zebrafish mutants die at day 8 post-fertilization, possibly because their development energy demands cannot be met, resulting in embryonic lethality. Some limitations to consider of the zebrafish experiments are that we did not perform rescue experiments to unequivocally demonstrate the specificity of our sgRNA designed to edit the Dhx15 gene by CRISPR/Cas9. However, we believe we can mitigate concerns about off-target effects considering the use of bioinformatically validated gRNAs with minimal off-target potential, as we show in Supplementary Figure S5. Another limitation to consider is the possibility that impaired liver organogenesis could contribute to the observed downregulation of Mypt1, Prox1, and Hdac3. However, the expression patterns of these factors, along with the overexpression of GATA6, which plays a significant role in liver organogenesis, suggest that the absence of the liver is not the primary cause of the observed differential expression of Mypt1, Prox1, and Hdac3 in Dhx15-deficient zebrafish.

In mammals, the embryonic liver is an early hematopoietic organ; therefore, mutations affecting liver or blood development may cause early lethality during embryogenesis [32,33]. In mice, hepatogenesis begins with the formation of the liver bud around gestation day 8.25 [34,35]. As we previously described, our *Dhx15*^−/−^ mice died prior to the embryonic stage E8.5 [7]. Therefore, we cannot disregard the possibility that embryonic lethality is linked to an impaired liver organogenesis caused by *Dhx15* deficiency. Due to early embryonic mortality associated with the loss of Dhx15, this hypothesis could only be tested using *Dhx15* conditional KO mice. In mice, RNA helicase knockout often results in embryonic lethality, implying their essential role in developmental processes [36,37,38]. For instance, the loss of *Ddx3x*, which is associated with cell survival and cell cycle control, causes early post-implantation lethality prior to E6.5 [39]. As far as we know, no other RNA helicase has been described to play an essential role in liver development.

Knowing that the vasculature is crucial in liver development, we studied the vascular phenotype in the liver of heterozygous mice. We had previously observed vascular defects in *Dhx15*^−/−^ zebrafish during development [7]. Here, we observed differences in thickness and connectivity of liver blood vasculature in *Dhx15*^+/−^ mice. Additionally, in *Dhx15*^+/−^ mice, we observed a significant reduction in *Angiopoietin 1*, *Vegf-c,* and *Podoplanin,* which play major roles in the blood and lymphatic vascular growth and maturation. These liver vasculature alterations suggest that Dhx15 might be affecting the development of the hepatic vascular network that is crucial in embryonic stages for the development of the liver and, as a result, altering liver organogenesis, as previously reported by others [40,41].

Vascular alterations within the liver might alter hepatic functionality. One of the main characteristics of the liver is its capacity to regenerate owing to the proliferative potential of quiescent hepatocytes. It was previously described that loss of Akt hinders hepatic regeneration by reducing cell proliferation, cell hypertrophy, glycogenesis, and lipid droplet formation [18]. Since Dhx15 is a downstream target of Akt 1 [6], we evaluated the effects of *Dhx15* depletion in liver regeneration. In this context, we observed decreased overall regeneration and cellular proliferation in *Dhx15*^+/−^ mice. However, the slightly decreased proliferation 48 h after hepatectomy was not linked to a decreased expression of proliferative genes such as Pcna or Cyclin d1. We also observed a lower mice survival after 72 h post-PHx. One of the reasons behind heterozygous mouse mortality at 72 h post-PHx might be correlated with a significantly reduced proliferation that is linked to a decreased expression of Cyclin d1.

We also studied an alternative mechanism to explain impaired function after partial hepatectomy. One of the major functions of the liver is to act as a “glucostat”. It has been demonstrated how glucose supplementation in liver regeneration mouse models increases survival in different gene deficiency models [42]. Furthermore, we had previously observed, by -omic analysis, that endothelial cells with a Dhx15-deficient background showed impaired glucose metabolism [7]. Therefore, we analyzed if *Dhx15* partial deficiency was promoting metabolic alterations. First, we observed that heterozygous mice were not able to normalize glycogen levels after partial hepatectomy. Such impaired glycogen restorage was linked to alterations in the expression of the enzymes of the glycogenic pathway such as glucokinase and glycogen synthase. During the regenerative process, glycogen is consumed and used as source of energy to meet with the high metabolic demands of regeneration [22,23]. This may indicate that the observed liver regeneration alterations may also be the outcome of a decreased energetic availability, hindering proper proliferation. In *Dhx15*^+/−^ mice, we also observed a lower glucose production. Accordingly, we show a significantly decreased glucose production in silenced hepatocytes that may be related to a reduced expression of *G6pc*, *Pklr*, and *Pc,* which are key enzymes needed for the release of free glucose into blood circulation. The metabolic defects caused by Dhx15 deficiency, together with the reduced cellular expression of regulators of the cell cycle progression, such as Cyclin d1, may explain the lower cell proliferation of hepatocytes found in *Dhx15*^+/−^ livers. These results combined, with the endothelial cell defects that surge at 72 h post PHx, result in an impaired liver regeneration derived from *Dhx15* partial deficiency.

Next, we evaluated the role of Dhx15 in the context of hepatocellular carcinoma (HCC) and liver metastasis. Recently, several studies have analyzed the role of DHX15 in different cancer types. DHX15 contributes as a cancer promoter in breast cancer, prostate cancer, acute myeloid leukemia, and hepatocellular carcinoma, and as an antitumor factor in glioma due to its growth inhibitory function [12,13,14,15,16]. In the present study, we evaluated the potential predictive use of DHX15 serologic analysis as a biomarker of HCC. Dhx15 in the serum of the HCC cohort was noticeably higher than that of healthy individuals. However, we did not detect significant differences in the Dhx15 circulating levels when we compared the HCC and the cirrhosis groups, despite detecting a trend of greater concentration of Dhx15 in patients with HCC. One important limitation of our results is the design of this observational clinical study. Therefore, and considering the urgent need of accurate biomarkers for HCC in the clinical laboratory, we guaranteed further prospective validation studies to confirm the diagnostic utility of DHX15 as a noninvasive biomarker for HCC in comparison with other liver tumors.

In a murine HCC xenograft model, we observed the formation of similar-volume primary tumors in WT and *Dhx15*^+/−^ mice. However, significantly smaller, and fewer tumoral nodules implanted in the liver were linked to Dhx15 depletion. In agreement with the reduced tumoral liver invasion, we observed a dysfunctional lymphatic vasculature in *Dhx15*^+/−^ mice. In a previous study modeling pulmonary metastasis in mice, we detected a significant metastasis reduction due to Dhx15 depletion [7]. Considering the role played by lymphatic vessels in tumor invasion [43], we suggest, as a model, that the impaired lymphatic growth within the primary tumor associated with Dhx15 deficiency limited cancer cell invasion into other organs, including the liver. These new findings highlight the role of Dhx15 as a potential target in metastasis.

To start evaluating the potential therapeutic benefits of Dhx15 inhibition in cancer and metastasis, we designed specific *Dhx15*-inhibiting oligonucleotides for in vivo use. We first analyzed the silencing potential of the AUM*silence* oligonucleotides in mouse and observed a successful Dhx15 inhibition in the liver and other organs. AUM*silence* ASOs are third-generation chemically modified oligos which have the capability of self-delivery (gymnosis) without the use of any delivery reagents or formulations. In our experience, AUM*silence* oligos are highly sequence specific to their target and show no toxicity. We have shown optimal delivery to the liver using AUM*silence* ASOs. Such attributes make AUM*silence* ASOs ideal for target discovery and preclinical studies. Then, we established the HCC Hepa 1-6 model in AUMsilence ASO-injected mouse to target *Dhx15* expression and we observed a significant reduction in average tumor growth. Evaluation of chronic toxicity, routes of administration, and dosage are still pending to establish robust conclusions about the biosafety of the AUMsilence ASO Dhx15 treatment. Also, liver-specific targeting of Dhx15 silencing and the generation of a conditional knockout to restrict the Dhx15 deficiency to the liver are needed to ensure the safety of an anti-Dhx15 therapeutic strategy in the context of tumor treatment and to prevent side effects in other organs. These experiments are currently underway in our laboratory.

We believe the presence of the other *Dhx15* allele in heterozygous mice prevents us from seeing a net effect on the decrease in the primary tumor volume. We base this rationale on the observation that silencing *Dhx15* in mice with the AUMsilence ASO Dhx15 treatment does have a clear impact on reducing the size of the primary tumor, since these specific anti-*Dhx15* oligo sequences further reduce the presence of Dhx15 compared to the levels of Dhx15 expression found in the *Dhx15*^+/−^ mice. Therefore, the absence of *Dhx15*, either in heterozygosis or due to specific *Dhx15* silencing, translates into an antitumor effect when analyzing tumor invasion or the size of the primary tumor. These concordant results achieved using two different experimental situations (gene deficiency and *Dhx15* silencing) robustly support our hypothesis that a potential antitumor strategy can be achieved by suppressing the activity of Dhx15. These results, in turn, agree with the role of DHX15 in tumor promotion discussed in the introduction [12,13,14,15,16] since, in these situations, there is a complete allelic expression of *DHX15*.

In summary, our study provides insights into an essential role for Dhx15 in the development of liver in zebrafish. Dhx15 knockout in zebrafish results in a liverless phenotype and early embryonic lethality. An impaired liver development could be the consequence of the observed blood and lymphatic vascular defects together with the reduced expression of hepatogenic enzymes. Also, Dhx15 depletion resulted in hepatic regeneration and metabolic alterations. The observed alterations in liver regeneration may be caused by a reduced proliferation linked to an aberrant glucose metabolism acting together with endothelial defects, impeding the de novo formation of hepatic vasculature and impaired liver regeneration. Also, Dhx15 deficiency led to reduced hepatic tumor invasion and tumor growth in a murine HCC model. Regarding the potential use of DHX15 as a diagnostic marker for liver disease, HCC patients showed increased levels of DHX15 in blood samples compared with subjects without hepatic affectation. Therefore, our results support the potential role of DHX15 as a diagnostic and therapeutic target in liver disease, as well as a major regulator of liver regeneration and organogenesis.

## 4. Materials and Methods

### 4.1. Mouse-Induced Tumor Model

HEPA 1-6 cells (ATCC, Manassas, VA, USA) were cultured in Dulbecco’s Modified Eagle Medium (DMEM) supplemented with 10% fetal calf serum, 50 U/mL penicillin, and 50 µg/mL streptomycin in humified atmosphere at 37 °C and 5% CO_2_. Syngeneic Hepa 1-6 tumor cells (5 × 10^6^) were subcutaneously injected into the flank of *Dhx15*^+/−^ and wild-type mice (n = 10). Primary tumor growth was controlled during the first 5 weeks. Tumor growth was monitored by measuring volumes using a digital slide-caliper. Tumor volume was calculated by the following formula: V = 4/3 × π × (length × depth × width). Primary tumors were fixed in 4% PFA and cryopreserved in tissue-tek O.C.T. compound (Sakura, Flemingweg, The Netherlands). The post-surgical metastasis model was performed as follows: five weeks post-injection, Hepa 1-6-injected mice were sacrificed, and the liver was extracted to perform metastasis analyses. Tile scan images of hematoxylin–eosin (H&E)-stained paraffin liver sections were visualized using an Olympus BX51 microscope equipped with DP71 camera (Olympus Europa SE & CO.KG., Hamburg, Germany), and the percentage of hepatic metastatic area as percent of total hepatic area was measured with ImageJ software (ImageJ version 1.52b; National Institutes of Health, Bethesda, MD, USA).

### 4.2. In Vivo Knockdown Experiments

Knockdown experiments were performed using AUM*silence^TM^* antisense oligonucleotides (AUM*silence^TM^* ASOs) that were designed and provided by AUM BioTech, LLC, Philadelphia, PA, USA. For general knockdown, the oligos were intravenously injected into the mouse tail vein at a dose of 10 mg/kg/day every third day. In vivo knockdown efficiency was determined by Dhx15 Western blot determination in different vital organs (liver, spleen, kidney, lungs, and heart), achieving an 80% knockdown in the liver. To study the impact of *Dhx15* depletion in the Hepa 1-6 HCC model, Hepa 1-6 cells (5 × 10^6^ cells, subcutaneously injected) were implanted subcutaneously into the flank of the mice (n = 10) and allowed to grow for five weeks in previously AUM*silence* ASO-injected mouse. An unrelated AUM*scramble* ASO SCR was used as a control. Tumor growth was monitored by measuring volumes using a digital slide-caliper.

### 4.3. Patients

A prospective cohort of consecutive patients treated at Hospital Clinic de Barcelona was evaluated. Informed consent was obtained from all patients involved in the study that was conducted according to the guidelines of the Declaration of Helsinki and approved by the Institutional Review Board of Hospital Clínic de Barcelona.

Included population consisted of patients with (1) HCC diagnosed according to AASL guidelines; (2) cirrhosis associated with hepatitis C virus infection (HCV) without HCC; and healthy volunteers. The demographic and clinical characteristics of the patients included in the study are shown in Supplementary Tables S1 and S2. This study included a total of 24 serum samples from control subjects without neoplastic or liver disease. The samples were collected, anonymized, and stored according to the ethical rules of the Hospital Clinic.

### 4.4. Statistical Analysis

Quantitative data were analyzed using GraphPad Prism 5 (GraphPad Software, Inc., San Diego, CA, USA), and statistical analysis of the results was performed using unpaired Student’s *t*-tests and ANOVA models (with Tukey’s post hoc test) with normally distributed data. Partial hepatectomy mortality scores were analyzed by log-rank test and survival curves were generated using the product limit method of Kaplan and Meier. For other type of data, the Mann–Whitney U-test was used. Correlations between variables were evaluated using Spearman’s rho or Pearson’s r, when appropriate. Differences were considered significant at a *p*-value < 0.05. The data are presented as the mean ± standard error of the mean.

## Data Availability

All data generated or analyzed during this study are included in this published article and its Supplementary Information files including a table with a list of all antibodies used in the study (Table S3).

## Supplementary Materials

The following supporting information can be downloaded at: https://www.mdpi.com/article/10.3390/ijms25073716/s1. References [44,45] are cited in the supplementary materials.

## References

[1] Studer M.K., Ivanović L., Weber M.E., Marti S., Jonas S. (2020). Structural basis for DEAH-helicase activation by G-patch proteins. Proc. Natl. Acad. Sci. USA.

[2] Silverman E., Edwalds-Gilbert G., Lin R.J. (2003). DExD/H-box proteins and their partners: Helping RNA helicases unwind. Gene.

[3] Sloan K.E., Bohnsack M.T. (2018). Unravelling the Mechanisms of RNA Helicase Regulation. Trends Biochem. Sci..

[4] Robert-Paganin J., Réty S., Leulliot N. (2015). Regulation of DEAH/RHA helicases by G-patch proteins. BioMed Res. Int..

[5] Abdelkrim Y.Z., Banroques J., Tanner N.K. (2021). RNA Remodeling Proteins: Methods and Protocols. Chapter 3: Known Inhibitors of RNA Helicases and Their Therapeutic Potential. Methods in Molecular Biology.

[6] Lee M.Y., Luciano A.K., Ackah E., Rodriguez-Vita J., Bancroft T.A., Eichmann A., Simons M., Kyriakides T.R., Morales-Ruiz M., Sessa W.C. (2014). Endothelial Akt1 mediates angiogenesis by phosphorylating multiple angiogenic substrates. Proc. Natl. Acad. Sci. USA.

[7] Ribera J., Portolés I., Córdoba-Jover B., Rodríguez-Vita J., Casals G., la Presa B.G.-D., Graupera M., Solsona-Vilarrasa E., Garcia-Ruiz C., Fernández-Checa J.C. (2021). The loss of DHX15 impairs endothelial energy metabolism, lymphatic drainage and tumor metastasis in mice. Commun. Biol..

[8] Lu H., Lu N., Weng L., Yuan B., Liu Y.-J., Zhang Z. (2014). DHX15 Senses Double-Stranded RNA in Myeloid Dendritic Cells. J. Immunol..

[9] Wang Y., He K., Sheng B., Lei X., Tao W., Zhu X., Wei Z., Fu R., Wang A., Bai S. (2021). The RNA helicase Dhx15 mediates Wnt-induced antimicrobial protein expression in Paneth cells. Proc. Natl. Acad. Sci. USA.

[10] Steimer L., Klostermeier D. (2012). RNA helicases in infection and disease. RNA Biol..

[11] Seiler M., Peng S., Agrawal A.A., Palacino J., Teng T., Zhu P., Smith P.G., Buonamici S., Yu L., The Cancer Genome Atlas Research Network (2018). Somatic Mutational Landscape of Splicing Factor Genes and Their Functional Consequences across 33 Cancer Types. Cell Rep..

[12] Ito S., Koso H., Sakamoto K., Watanabe S. (2017). RNA helicase DHX15 acts as a tumour suppressor in glioma. Br. J. Cancer.

[13] Mosallanejad K., Sekine Y., Ishikura-Kinoshita S., Kumagai K., Nagano T., Matsuzawa A., Takeda K., Naguro I., Ichijo H. (2014). The DEAH-Box RNA Helicase DHX15 Activates NF-κB and MAPK Signaling Downstream of MAVS During Antiviral Responses. Sci. Signal..

[14] Pan L., Li Y., Zhang H.Y., Zheng Y., Liu X.L., Hu Z., Wang Y., Wang J., Cai Y.H., Liu Q. (2017). DHX15 is associated with poor prognosis in acute myeloid leukemia (AML) and regulates cell apoptosis via the NF-kB signaling pathway. Oncotarget.

[15] Jing Y., Nguyen M.M., Wang D., E Pascal L., Guo W., Xu Y., Ai J., Deng F.-M., Masoodi K.Z., Yu X. (2018). DHX15 promotes prostate cancer progression by stimulating Siah2-mediated ubiquitination of androgen receptor. Oncogene.

[16] Xie C., Liao H., Zhang C., Zhang S. (2019). Overexpression and clinical relevance of the RNA helicase DHX15 in hepatocellular carcinoma. Hum. Pathol..

[17] Morales-Ruiz M., Cejudo-Martín P., Fernández-Varo G., Tugues S., Ros J., Angeli P., Rivera F., Arroyo V., Rodés J., Sessa W.C. (2003). Transduction of the liver with activated Akt normalizes portal pressure in cirrhotic rats. Gastroenterology.

[18] Pauta M., Rotllan N., Fernández-Hernando A., Langhi C., Ribera J., Lu M., Boix L., Bruix J., Jimenez W., Suárez Y. (2016). Akt-mediated foxo1 inhibition is required for liver regeneration. Hepatology.

[19] Morales-Ruiz M., Fondevila C., Muñoz-Luque J., Tugues S., Rodríguez-Laiz G., Cejudo-Martín P., Romero J.M., Navasa M., Fuster J., Arroyo V. (2007). Gene transduction of an active mutant of Akt exerts cytoprotection and reduces graft injury after liver transplantation. Am. J. Transplant..

[20] Zhao M., Ying L., Wang R., Yao J., Zhu L., Zheng M., Chen Z., Yang Z. (2021). DHX15 Inhibits Autophagy and the Proliferation of Hepatoma Cells. Front. Med..

[21] McElderry J., Carrington B., Bishop K., Kim E., Pei W., Chen Z., Ramanagoudr-Bhojappa R., Prakash A., Burgess S.M., Liu P.P. (2019). Splicing factor DHX15 affects tp53 and mdm2 expression via alternate splicing and promoter usage. Hum. Mol. Genet..

[22] Murray A.B., Strecker W., Silz S. (1981). Ultrastructural changes in rat hepatocytes after partial hepatectomy, and comparison with biochemical results. J. Cell Sci..

[23] Huang J., Rudnick D.A. (2014). Elucidating the metabolic regulation of liver regeneration. Am. J. Pathol..

[24] Stacker S.A., Caesar C., Baldwin M.E., Thornton G.E., Williams R.A., Prevo R., Jackson D.G., Nishikawa S.-I., Kubo H., Achen M.G. (2001). VEGF-D promotes the metastatic spread of tumor cells via the lymphatics. Nat. Med..

[25] Kalota A., Karabon L., Swider C.R., Viazovkina E., Elzagheid M., Damha M.J., Gewirtz A.M. (2006). 2′-Deoxy-2′-fluoro-β-D-arabinonucleic acid (2′F-ANA) modified oligonucleotides (ON) effect highly efficient, and persistent, gene silencing. Nucleic Acids Res..

[26] Farooq M., Sulochana K., Pan X., To J., Sheng D., Gong Z., Ge R. (2008). Histone deacetylase 3 (hdac3) is specifically required for liver development in zebrafish. Dev. Biol..

[27] Wallace K.N., Yusuff S., Sonntag J.M., Chin A.J., Pack M. (2001). Zebrafish hhex regulates liver development and digestive organ chirality. Genesis.

[28] Tao S., Witte M., Bryson-Richardson R.J., Currie P.D., Hogan B.M., Schulte-Merker S. (2011). Zebrafish prox1b mutants develop a lymphatic vasculature, and prox1b does not specifically mark lymphatic endothelial cells. PLoS ONE.

[29] Zhao R., Watt A.J., Li J., Luebke-Wheeler J., Morrisey E.E., Duncan S.A. (2005). GATA6 Is Essential for Embryonic Development of the Liver but Dispensable for Early Heart Formation. Mol. Cell Biol..

[30] Huang H., Ruan H., Aw M.Y., Hussain A., Guo L., Gao C., Qian F., Leung T., Song H., Kimelman D. (2008). Mypt1-mediated spatial positioning of Bmp2-producing cells is essential for liver organogenesis. Development.

[31] Anderson J.L., Carten J.D., Farber S.A. (2011). Zebrafish Lipid Metabolism: From Mediating Early Patterning to the Metabolism of Dietary Fat and Cholesterol. Methods Cell Biol..

[32] Reimold A.M., Etkin A., Clauss I., Perkins A., Friend D.S., Zhang J., Horton H.F., Scott A., Orkin S.H., Byrne M.C. (2000). An essential role in liver development for transcription factor XBP-1. Genes Dev..

[33] Tao T., Peng J. (2009). Liver development in zebrafish (*Danio rerio*). J. Genet. Genom..

[34] Gualdi R., Bossard P., Zheng M., Hamada Y., Coleman J.R., Zaret K.S. (1996). Hepatic specification of the gut endoderm in vitro: Cell signaling and transcriptional control. Genes Dev..

[35] Duncan S.A., Watt A.J. (2001). BMPs on the road to hepatogenesis. Genes Dev..

[36] Hildebrandt M.R., Germain D.R., Monckton E.A., Brun M., Godbout R. (2015). Ddx1 knockout results in transgenerational wild-type lethality in mice. Sci. Rep..

[37] Lee C.G., Soares V.D., Newberger C., Manova K., Lacy E., Hurwitz J. (1998). RNA helicase A is essential for normal gastrulation. Proc. Natl. Acad. Sci. USA.

[38] Janknecht R. (2010). Review Article Multi-talented DEAD-box proteins and potential tumor promoters: p68 RNA helicase (DDX5) and its paralog, p72 RNA helicase (DDX17). Am. J. Transl. Res..

[39] Chen C.-Y., Chan C.-H., Chen C.-M., Tsai Y.-S., Tsai T.-Y., Lee Y.-H.W., You L.-R. (2016). Targeted inactivation of murine Ddx3x: Essential roles of Ddx3x in placentation and embryogenesis. Hum. Mol. Genet..

[40] Gouysse G., Couvelard A., Frachon S., Bouvier R., Nejjari M., Dauge M.C., Feldmann G., Hénin D., Scoazec J.Y. (2002). Relationship between vascular development and vascular differentiation during liver organogenesis in humans. J. Hepatol..

[41] DeSesso J.M. (2017). Vascular ontogeny within selected thoracoabdominal organs and the limbs. Reprod. Toxicol..

[42] Fernández M.A., Albor C., Ingelmo-Torres M., Nixon S.J., Ferguson C., Kurzchalia T., Tebar F., Enrich C., Parton R.G., Pol A. (2006). Caveolin-1 Is Essential for Liver Regeneration. Science.

[43] Alitalo A., Detmar M. (2012). Interaction of tumor cells and lymphatic vessels in cancer progression. Oncogene.

[44] Cermak T., Doyle E.L., Christian M., Wang L., Zhang Y., Schmidt C., Baller J.A., Somia N.V., Bogdanove A.J., Voytas D.F. (2011). Efficient design and assembly of custom TALEN and other TAL effector-based constructs for DNA targeting. Nucleic Acids Res..

[45] Higgins G.M., Anderson R.M. (1931). Experimental pathology of the liver. I. Restoration of the liver of the white rat following partial surgical removal. Arch. Pathol..

