# Supplementary material for: Identification of Dhx15 as a Major Regulator of Liver Development, Regeneration, and Tumor Growth in Zebrafish and Mice
Source: Int J Mol Sci. 2024 Mar 27;25(7):3716. doi: 10.3390/ijms25073716 (PMC11011938; doi:10.3390/ijms25073716)
Supplement: Supplementary file 1 [file ijms-25-03716-s001.zip › ijms-2912322-supplementary.pdf]

Supplementary file - Identification of Dhx15 as a major regulator of liver development, regeneration and tumor growth in zebrafish and mice

Table of content:

1. Additional Materials and Methods
2. Additional Figures and Legends
3. Additional Tables
4. Additional References

ADDITIONAL MATERIALS AND METHODS

Generation of the Zebrafish animal model. Adults wild-type zebrafish (*Danio rerio*), in a Tg(*flk1*:EGFP);Tg(*fabp10*:RFP) background, purchased from KIT -European Zebrafish Resource Center (EZRC), were maintained at 28–29 ºC on a light cycle of 14 hours light/10 hours dark. The Crispr/cas9 design for gene knock-out was performed as follows: Gene sequences were retrieved from http://www.ncbi.nlm.nih.gov/gene and http://www.ensembl.org/Danio_rerio/Info/Index. The sgRNA was designed using the online tool http://crispor.tefor.net, based on exon site and high efficacy and off-target published algorithms. We designed specific sgRNA (gaggctgcagttgcccgtctggg) targeting

the genomic locus on the second exon which corresponds to the first coding exon of the *Dhx15* gene (Supplementary Fig. 4A). The *in silico* evaluation of the sgRNA sequence revealed a total of 13 off-targets. None located in an exon area, and only one of these potential off-target sequences was situated on the same chromosome as the gene of interest. Therefore, we estimated the probability of having off-targets as low. The sgRNA was assembled in vitro with Cas9 endonuclease and microinjected in one-cell stage embryos. Fertilized zebrafish embryos were collected in E3 medium. Then, embryos were grown at 28.5ºC. The mutagenesis efficacy was evaluated on pools of 30 injected embryos, whose genomic DNA was amplified by PCR around the DNA break site and analyzed via T7 endonuclease system. This strategy allowed us to select injected pools to grow to adulthood both in F0 and F1 generations. When F1 animals reached adulthood, their fins were individually clipped and used to extract genomic DNA. The region of interest was amplified and analyzed by Sanger sequencing to identify isogenic mutant animals promoting the appearance of early stop codons in *Dhx15*. As shown in Supplementary Fig. 4B, a deletion of 4 bp is generated in the mutant strand. This leads to the appearance of an early stop codon in the mutant strand (black box aminoacid; right panel Supplementary Fig. 4B).

Dhx15 transgenic mice. *Dhx15* gene deficient C57BL/6 mice were generated by genomic editing by microinjecting TALEN (transcription activator-like effector nuclease technology) RNA in pronucleated oocytes (Cyagen). The mouse *Dhx15* gene (GenBank accession number: NM_007839.3; Ensembl: ENSMUSG00000029169) is located on chromosome 5. Fourteen exons have been identified for this gene, with the ATG start codon in exon 1 and the TGA stop codon in exon 14. Exon 2 was selected as TALEN

target sites. TALENs were constructed using the Golden Gate Assembly method [1] and confirmed by sequencing. The amplicons were then purified and sent for DNA sequencing analysis. TALEN mRNAs generated by in vitro transcription were injected into fertilized eggs for knockout mouse production (cDNA sequence: TGTTGGTGAGACTGGGTC). The pups were genotyped by PCR followed by sequence analysis. The positive founders were breeding to the next generation, which was genotyped by PCR and DNA sequencing analysis. DNA sequencing revealed two different *Dhx15*-deficient mouse clones: Mouse-ID#35 that was missing 8 bases in one strand, and Mouse-ID#39 that was missing 1 base in one strand. Wild-type DNA was used as a negative control for sequencing in parallel. The mRNA transcribed from targeted allele with frameshift undergoes nonsense-mediated decay (NMD). All animals were kept under constant temperature and humidity in a 12-hour controlled dark/light cycle, and they were fed *ad libitum* on a standard pellet diet. All mice studied in the experimental protocols were aged between 3-4 months. Also, wild-type and *Dhx15* gene deficient female and male mice were used indistinctively in a percentage of nearly 50% for every gender. We did not find significant statistical differences attributable to the gender variable. We performed the study following the guidelines of the Investigation and Ethics Committees of the Hospital Clínic and the University of Barcelona.

Mouse genotyping. Mouse genomic DNA was isolated from tail biopsies using a specific kit (Extract-N-Amp™ Tissue PCR Kit; Sigma-Aldrich, Darmstadt, Germany). PCR was performed using the primer pairs to amplify the *Dhx15* gene (primer forward: 5′CACCAACCTGCCCCATACTCCT-3′ and primer reverse: 5′-TGTATTGTCCCAGGGTAAAATGTGTTG-3′). PCR conditions were as follows: 35

cycles at 94°C for 30 s, 59.3°C for 30 s, and 72°C for 60 s. PCR product was sequenced by sanger sequencing to distinguish the *Dhx15* wild type mice and *Dhx15* transgenic mice.

DHX15 silencing in hepatocytes. The silencing of *Dhx15* was carried out in mouse primary hepatocytes immortalized with the SV40 virus (HEP; abmGood, Richmond, Canada), through shRNA by lentiviral infection (Dharmacon, Lafayette, Colorado, USA). The SMARTvector incorporated the bipartite 3G Tet-On® induction system, an inducible system with minimal basal expression and potent activation after induction with doxycycline. Cells were cultured in Complete Hepatocyte Medium (Pelobiotech, Planegg, Germany) in humified atmosphere at 37°C and 5% CO_2.

Zebrafish hepatic characterization. Tg(*flk1*:EGFP);Tg(*fabp10*:RFP) zebrafish express Red Fluorescent Protein (RFP) in all liver cells. At 3 hours post fertilization (hpf) abnormal or not fertilized embryos are discarded, and embryos develop until 128 hpf at 28.5ºC, when the liver is fully developed. At 128 hpf, embryos are fixed in 4% paraformaldehyde for 2-4h at room temperature (RT) and then washed 3x with PBS. Fixed larvae are observed under a fluorescence stereo microscope (Olympus MVX10) and photographed with a digital camera (Olympus DP71) and cell'D software. RFP filtered images are taken and then analyzed using the FIJI software for hepatomegaly and necrosis assessment. Afterwards, steatosis and yolk retention are evaluated by Oil Red O staining. Fixed larvae are bleached using Bleach solution for 20 minutes at RT and then washed 5x with PBS. Bleached embryos are first submerged in 85% Propylene glycol (PG) for 10 minutes and then in 100% PG for another 10 minutes before staining with Oil Red O o.5% in 100% PG for 30 minutes, 50 minutes in 85% PG

and 40 minutes in 85% PG with equal volume of PBS. Finally, embryos are washed 1x with PBS before adding 80% glycerol. Bright field images are then taken to detect both steatosis and yolk retention. For steatosis assessment, larvae are considered to be positive when 3 or more round lipid droplets are visible within the hepatic parenchyma.

Partial hepatectomy surgical procedure. All surgeries were carried out under isoflurane (Sigma-Aldrich, St. Louis, MO) anesthesia. Two-thirds partial hepatectomy (PHx) was performed according following Higgins and Johnson technique [2]. The abdomen was opened with a midline incision. Two-thirds of the liver (median and left lobes) was removed. After PHx, wildtype (WT) mice (n=7) and *Dhx15*$^{+/-}$ mice (n=8) were sacrificed at different time points: 2, 3 and 7 days. The regenerating bottom right lobe was snap-frozen in liquid nitrogen, and the upper right lobe was either fixed in 4% paraformaldehyde at 4ºC, cryoprotected overnight in 30% sucrose solution, and embedded in optimal cutting temperature medium (Tissue-Tek O.C.T. Compound; Sakura) or fixed in 10% buffered formaldehyde and embedded in paraffin for future processing. The percentage of liver regeneration was calculated by the following formula: Weight of non-removed lobes/Total body weight of mice (Higgins Index). Daily monitoring after hepatectomy allowed the detection of deceased mice.

Immunostaining Assays. For the immunofluorescence assay, tissues were fixed in 4% paraformaldehyde, cryoprotected overnight in a 30% sucrose solution, and embedded in optimal cutting temperature medium. Next, 3 μm frozen sections were rehydrated, blocked with 5% normal goat serum, and incubated with rabbit anti-mouse Ki-67 (1:100; Abcam), rabbit anti-mouse ERG (1:100; Cell Signaling), rat anti-mouse endomucin (1:100; Abcam), anti-mouse Lyve-1 (1:100; Abcam). The binding sites of the primary

antibody were revealed with Alexa Fluor 488-conjugated donkey anti-rabbit IgG (Invitrogen), Alexa Fluor 555-conjugated donkey anti-rabbit IgG (Invitrogen) or Alexa Fluor 488-conjugated rabbit anti-rat IgG (Invitrogen). Cells were counterstained with 40,6-diamidino-2-phenylindole to visualize the nuclei. Stainings were analyzed using an Olympus BX51 microscope equipped with DP71 camera (Olympus Europa SE & CO.KG. Germany). The quantification of the number of hepatic vessels was performed in 10 randomly selected microscopic fields obtained from the liver of each mouse. The average value obtained from the 10 counts was subsequently used for statistical analysis. Five randomly selected microscopic fields per mouse were used to quantify the average value of the length of the internal vascular perimeter of hepatic blood vessels. This parameter was measured by computer-assisted morphometry using the Segmented Line Tool available in the ImageJ software (ImageJ version 1.37; National Institutes of Health, Bethesda, MD, USA).

Liver cryosections images from rabbit anti-Pecam1 (1:100; Cell Signalling) stained tissue were obtained using a laser scanning confocal spectral microscope Leica TCS SP5 (Leica Microsystems Heidelberg GmbH, Manheim, Germany).

Western Blot analysis. Tissue lysates were prepared in a lysis buffer (Tris–HCl 20 mM pH 7.4 containing 1% Triton X-100, 0.1% SDS, 50 mM NaCl, 2.5 mM EDTA, 1 mM $Na_4P_2O_7$ $10H_2O$, 20 mM NaF, 1 mM Na_3VO_4, 2 mM Pefabloc and Complete® from Roche). Proteins were separated on a 10% SDS-polyacrylamide gel (Mini Protean III; Biorad, Hercules, CA, US) and transferred for 2 hours at 4ºC to nitrocellulose membranes (Transblot Transfer Medium; Biorad, Hercules, CA, US) that were stained with Ponceau-S red as a control for protein loading. Membranes were incubated at 4ºC

overnight with the following antibodies: rabbit anti-Ccnd1 (1:1000; Cell Signaling), mouse anti-proliferating cell nuclear antigen (Pcna; 1:1000; Sigma), mouse anti-Dhx15 (1:200; Santa Cruz), and β-actin (1:1000; Sigma). Next, membranes were incubated with goat anti-rabbit peroxidase-conjugated secondary antibody or goat anti-mouse peroxidase-conjugated secondary antibody at a 1:2000 dilution (Cell Signaling, Danvers, MA, US) for 1 hour at room temperature. The bands were visualized by chemiluminescence (Clarity Western ECLS substrate; Biorad, Hercules, CA, US). The intensity of each band was quantified by Image J software (ImageJ version 1.52b; National Institutes of Health, Bethesda, MD, USA). Band intensities were measured and normalized to the indicated sample (shown as 1.00) on the same membrane.

Biochemical assays. Serum was obtained by blood centrifugation and biochemical parameters were assayed using an Atellica automated analyzer (Siemens Healthineers, Tarrytown, NY, USA). Intra-assay and inter-assay coefficient of variation were lower than 6% and 8%, respectively, in all cases. All the parameters were measured according to the manufacturer instructions. In these serum samples, albumin, bilirubin, blood urea nitrogen (BUN) and alpha-fetoprotein (AFP) were determined.

To determine liver gluconeogenesis, pyruvate tolerance tests were performed, mice were fasted for 24 h and injected intraperitoneally with 2mM sodium pyruvate dissolved in Ringer solution (Sigma). Blood glucose concentrations were measured at basal state (0 min) and 15, 30, 60, 90, and 120 min after the injection with the Lisubel Chek PLUS TD monitoring system (TaiDoc Technology Corporation, Taiwan). To determine intracellular glucose production, si-HepDhx15 and WT-Hep were cultured in P6-well plates. Cells were cultured in serum-free media for 2 hours, then 2mM pyruvate stimuli

was added. Cellular glucose production was measured with the High Sensitivity Glucose Assay Kit (Sigma) as per manufacturers' instructions. To determine glycogen concentrations in the liver, tissue homogenates, extracted at basal state and two days after hepatectomy, were used in the glycogen assay kit (Sigma) as per manufacturers' instructions.

Histology. Frozen sections of 8 μm were rehydrated and hepatic glycogen content was assessed in 8-μm liver sections that were embedded in paraffin and fixed in 10% buffered formaldehyde solution. Sections were periodic acid-Schiff-stained, according to manufacturer's instruction (Sigma- Aldrich), and counterstained with hematoxylin (Sigma-Aldrich). Total hepatocyte cell area was quantified in hematoxylin and eosin-stained liver sections. The cell areas in the photomicrographs were measured using the software ImageJ (version 1.37; National Institutes of Health, Bethesda, MD). Stainings were analyzed using an Olympus BX51 microscope equipped with DP71 camera (Olympus Europa SE & CO.KG. Germany).

Real-time quantitative reverse transcription PCR. Total RNA was extracted from frozen mouse livers and cultured si-Dhx15 hepatocytes with Trizol reagent (Life Technologies, Rockville, MD). One microgram of total RNA was reverse transcribed using the First Strand cDNA Synthesis Kit (Roche). Then, complementary DNA samples were amplified for 30–35 cycles (94°C for 30 s, 55–60°C for 30 s, 72°C for 1 min; 7900HT Fast Real-Time PCR System, Applied Biosystems). To normalize the results, the HPRT gene was used as a reference. Specific primers for amplification of the complementary DNA can be found in the Supplementary table 1.

Enzyme-linked immunosorbent assay (ELISA) of DHX15. Quantification of DHX15 levels in human serum was performed using specific ELISAs (MyBioSource) according to the manufacturer's instructions.

ADDITIONAL FIGURES AND LEGENDS

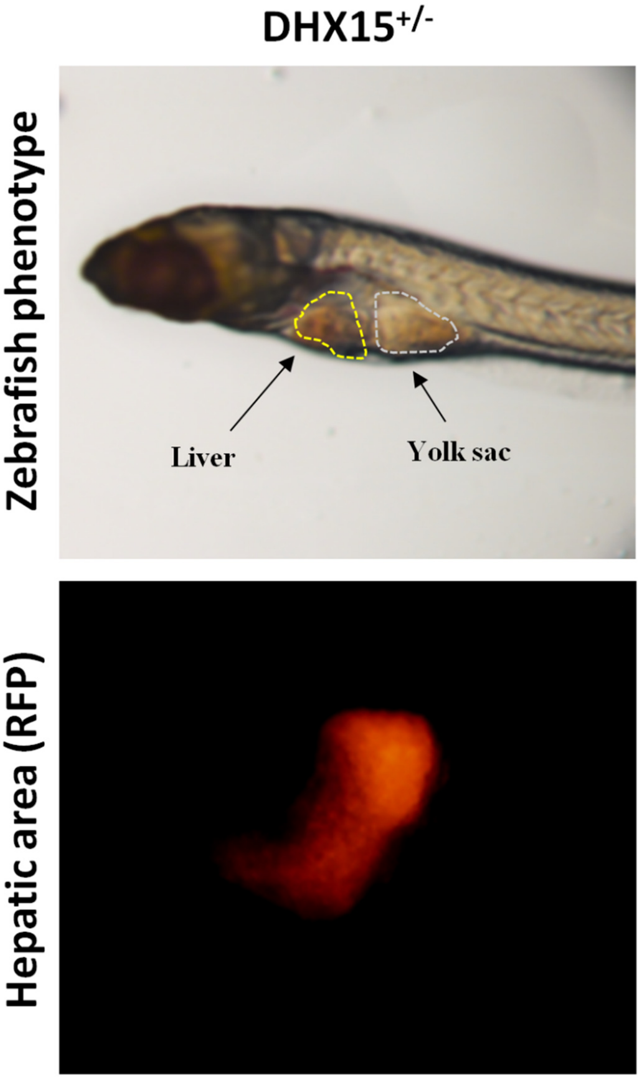

Supplementary figure S1. Heterozygous *Dhx15* deficient zebrafish embryos develop liver. On the upper panel, representative image of *Dhx15*$^{+/-}$ larvae at 5 day post fertilization (dpf) revealing proper liver development and yolk sac. Each area is enclosed with different colors (yellow lines correspond to liver region and green lines to yolk sac). On the lower panel, positive liver red fluorescence in *Dhx15*$^{+/-}$ larvae.

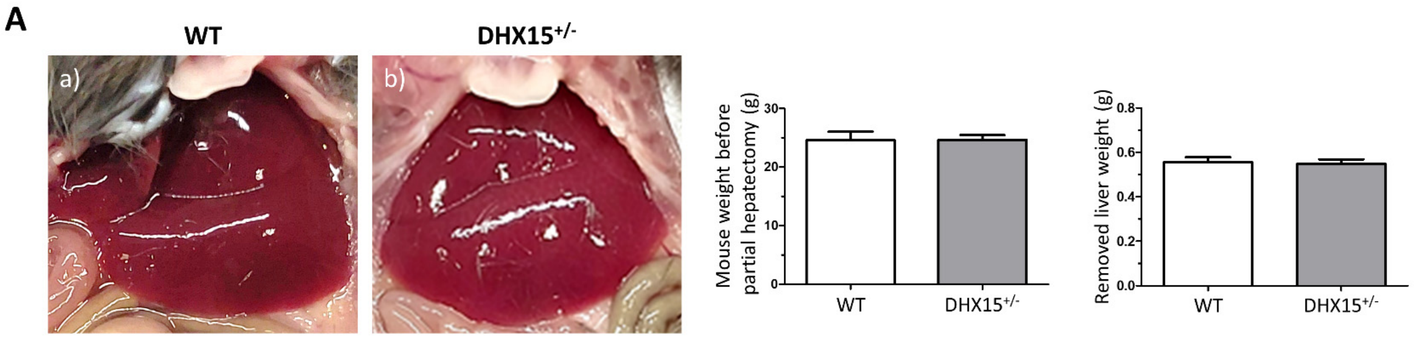

Supplementary figure S2. Heterozygous *Dhx15* deficient mice present normal liver size and function. (A) Representative macroscopic images of livers from wild-type (panel a) and $Dhx15^{+/-}$ (panel b) adult mice. Graphs on the right show basal mice weight and total liver weight, respectively (mean ± SEM; n=8). (B) Blood levels of albumin, bilirubin and blood urea nitrogen (BUN) in wild-type and $Dhx15^{+/-}$ mice (mean ± SEM; n=5).

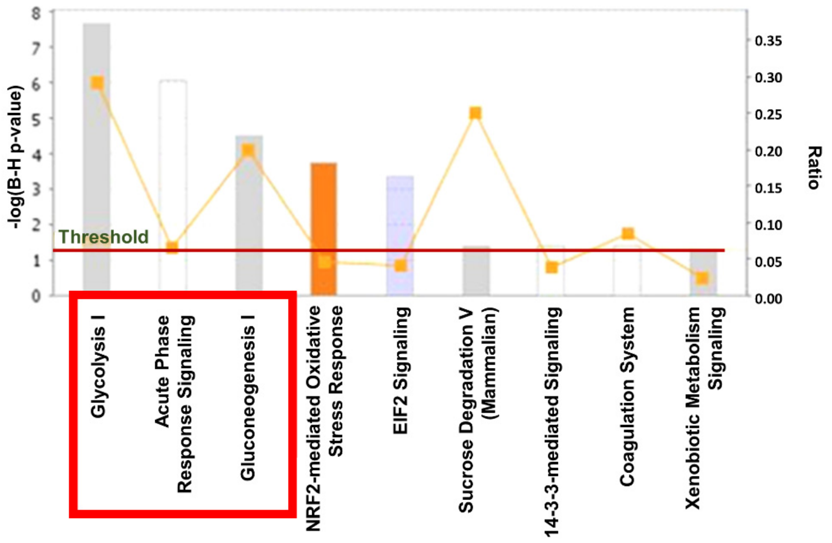

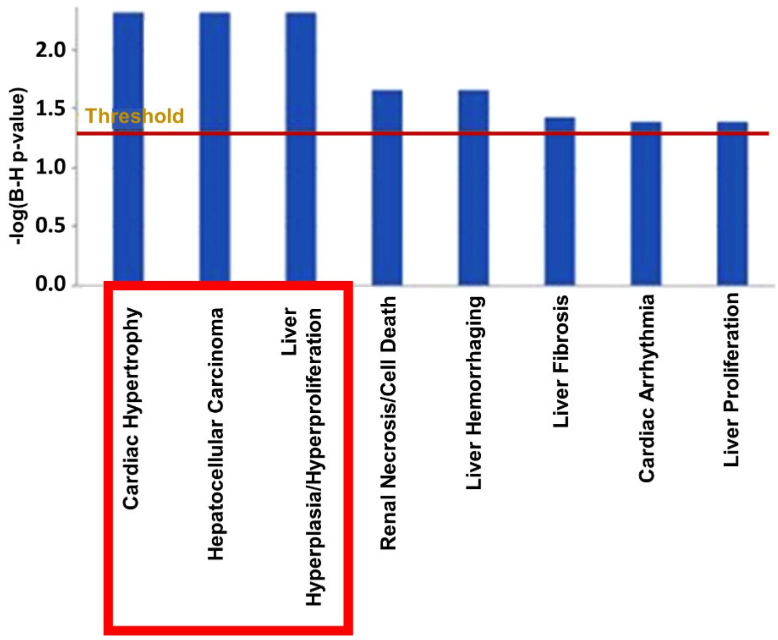

Supplementary figure S3. Bioinformatic analysis of the proteome and the transcriptome obtained from *Dhx15* silenced endothelial cells. Proteomic and transcriptomic analysis of wild-type endothelial cells versus endothelial cells with the

Dhx15 silenced revealed a differential expression of enzymes that participate in different metabolic processes, most of them related to the pathway of glycolysis/gluconeogenesis and liver dysfunction (n=5, for each experimental condition). In the upper graph, the comparison of canonical pathways affected by the knockout of Dhx15 activity in endothelial cells were modeled using the Ingenuity Pathways Knowledge Base (IPA). The results obtained were compared with global molecular networks using the Fisher's Exact Test. The resulting P-values were adjusted for multiple comparisons using the Benjamini and Hochberg's method to control the false discovery rate. After multi-test adjustment, differences were considered as significant at a P value less than 0.05 (left axis). The red line across the graph indicates the point where the significance value equals 0.05. In addition, the significance of the association was measured based on the ratio of the number of targets from the data set that map to the pathway divided by the total number of targets that are included in the canonical pathway (yellow line and right axis). In the lower graph, the comparison of diseases and biological functions affected by the knockout of Dhx15 activity in endothelial cells were modeled using the Ingenuity Pathways Knowledge Base (IPA). The results obtained were compared with global molecular networks using the Fisher's Exact Test. The resulting P-values were adjusted for multiple comparisons using the Benjamini and Hochberg's method to control the false discovery rate. After multi-test adjustment, differences were considered as significant at a P value less than 0.05. The red line across the graph indicates the point where the significance value equals 0.05.

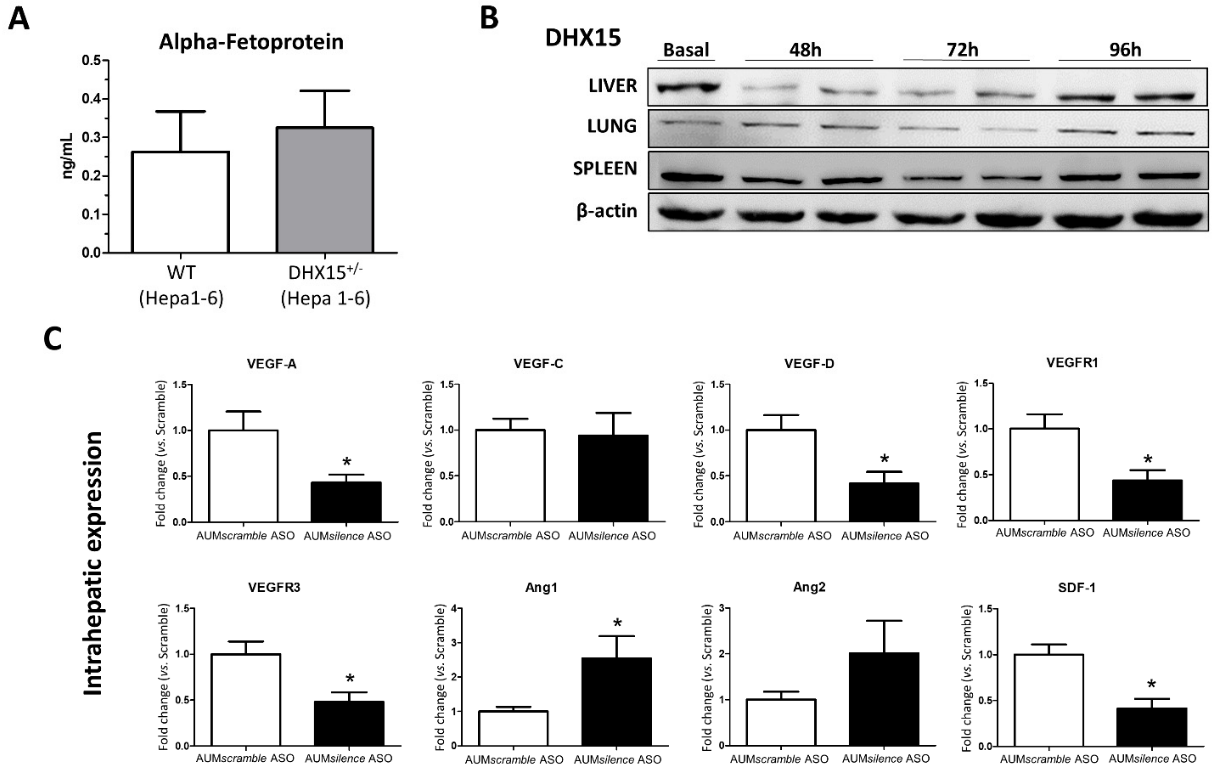

Supplementary figure S4. Tissue Dhx15 silencing after AUM*silence* ASO treatment.

(A) Blood levels of alpha-fetoprotein in wild-type and *Dhx15*$^{+/-}$ mice 5 weeks after implantation of mouse Hepa1-6 hepatoma cells (mean ± SEM; n=8). (B) Dhx15 protein expression in liver, lung and spleen was evaluated by western blot using tissue lysates from wild-type AUM*silence* ASO (*Dhx15* specific) -injected mice. β-actin was used as a loading control. (C) RNA extraction of liver tissue from wild-type AUM*silence* ASO (*Dhx15* specific) or AUM*scramble* ASO (scramble) injected mice was performed. mRNA expression was analyzed by RT-qPCR. Graphs show the expression levels of *Vegf-a*, *Vegf-c*, *Vegf-d*, *Vegfr1*, *Vegfr3*, *Angiopoietin 1*, *Angiopoietin 2* and *Sdf-1* genes in AUM*scramble* ASO and AUM*silence*-Dhx15 conditions. mRNA levels are shown as fold

change relative to *Hprt* mRNA levels. Bars represent mean ± SEM, *$p<0.05$ *vs.* scramble (n=4).

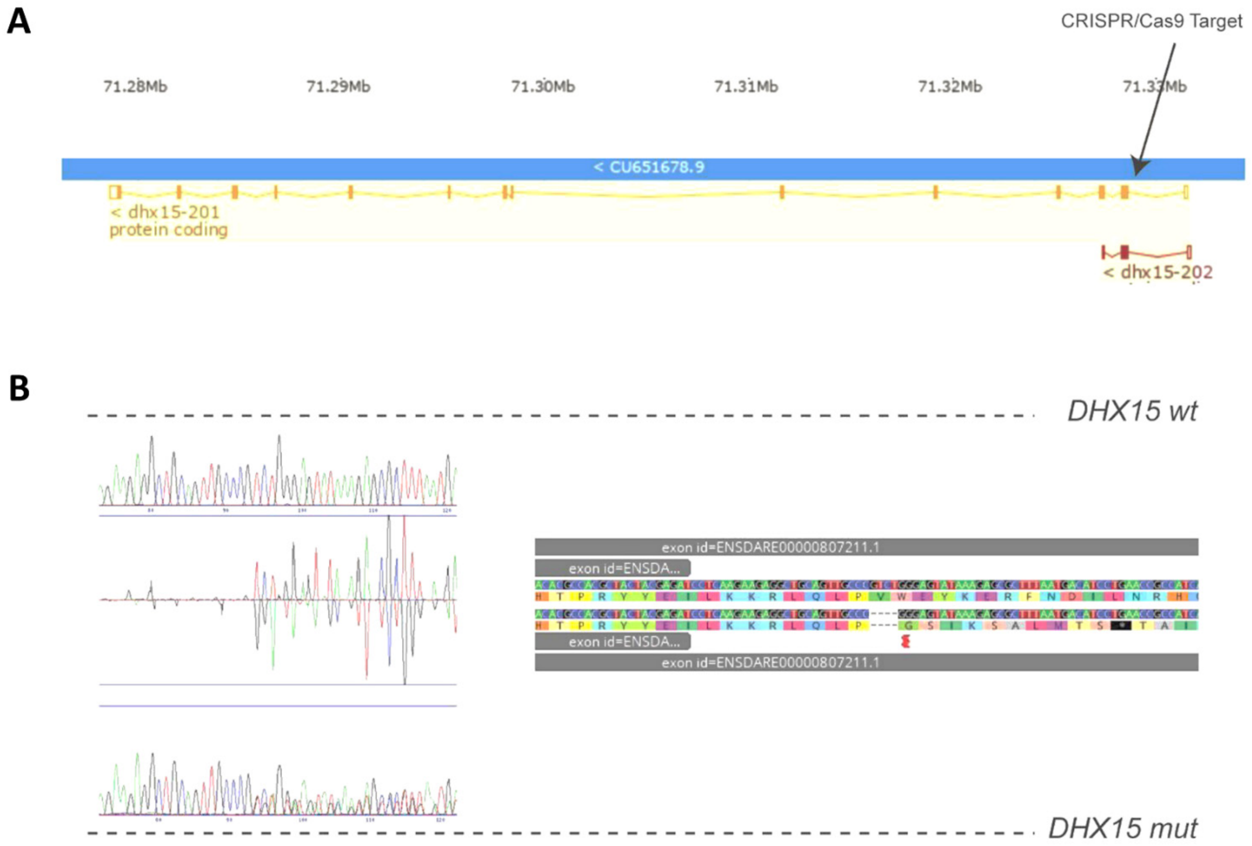

Supplementary figure S5. Schematic representation of *Dhx15* gene and CRISPR design strategy. (A) Zebrafish *Dhx15* genomic locus in chromosome 4 displaying all coding exons (yellow boxes). (B) Alignment of wild-type and mutant *Dhx15* sequence obtained by Sanger sequencing.

ADDITIONAL TABLES

Group	N	Age<br>Median (IQR)	Sex<br>% Male
Control	24	62.7 (59.0-71.5)	33.3%
Cirrhotic	35	60.2 (54.8-69.5)	68.6%
Hepatocellular carcinoma	62	66.9 (61.0-71.0)	70.0%
**Total**	**121**	**63.3 (60.0-71.0)**	**57.3%**

Table S1. Demographic characteristics of the patients included in this study.

Notes: Variables are expressed as median and interquartile range (IQR). N: number of patients in which it was possible to determine the parameter.

Parameters	Control<br>Median (IQR)	Cirrhotic<br>Median (IQR)	Hepatocellular carcinoma<br>Median (IQR)	Reference range
**Markers of liver injury and function**				
ASAT	24.7 (20.3-30.8)	29.0 (24.0-38.0)	**71.3 (37.5-93.5)**	5-40 IU/L
ALAT	24.6 (18.3-26.8)	26.0 (20.0-32.0)	**62.4 (32.0-81.5)**	5-40 IU/L
GGT	35.7 (20.0-35.0)	28.0 (18.0-48.0)	**309.0 (63.0-314.0)**	5-40 IU/L
Albumin	40.1 (41.5-44.5)	45.0 (41.0-47.0)	40.2 (37.0-45.0)	34-48 g/L
Bilirubin	0.6 (0.5-0.7)	0.8 (0.6-1.1)	1.1 (0.6-1.3)	< 1.2 mg/ dL
**Others**				
Glucose	97.0 (83.0-102.0)	101.0 (77.0-128.0)	**115.3 (89.3-131.3)**	65-110 mg/dL
Cholesterol	185.7 (166.3-195.3)	197.0 (133.0-212.0)	190.5 (134.0-233.0)	≤ 200 mg/dL
Triglycerides	95.4 (68.5-122.0)	142.0 (68.0-165.0)	125.9 (69.0-147.0)	< 150 mg/dL
Alfa-fetoprotein	3.9 (2.0-5.3)	4.0 (2.1-4.9)	**483.1 (3.3-245.0)**	< 10 ng/mL

Table S2. Analytical parameters determined in the serum of patients.

Notes: Variables are expressed as median and interquartile range (IQR). Values outside the range are in bold. IU: International Units, ALAT: alanine aminotransferase, ASAT: aspartate aminotransferase, GGT: gamma-glutamyl transferase.

Table S3. Experimental and commercial details of the antibodies used in the study.

Name	Supplier	Dilution
Rat anti-mouse **CD31**	BD pharmigen (550274)	1:100
Rabbit anti-mouse **Podoplanin**	Merck (P1995)	1:100
Rabbit anti-mouse **Lyve1**	Abcam (ab281587)	1:100
Rat anti-mouse **Endomucin**	Abcam (ab106100)	1:100
Rabbit anti-mouse **Ki67**	Abcam (ab15580)	1:100
Rabbit anti-mouse **CyclinD1**	Cell Signaling (#2922)	1:1000
Rabbit anti-mouse **ERG**	Cell Signaling (#97249)	1:100
Mouse anti-mouse **PCNA**	Cell Signaling (#2586)	1:1000
Rabbit anti-mouse **β-actin HRP**	Cell Signaling (#5125)	1:1000
Mouse anti-mouse **DHX15**	Santa Cruz Biotechnology (sc-271686)	1:200
Goat **anti-rabbit Alexa Fluor 555**	Thermo Fisher Scientific (A32732)	1:500
Goat **anti-rabbit Alexa Fluor 488**	Thermo Fisher Scientific (A11008)	1:500
Donkey **anti-rat Alexa Fluor 488**	Thermo Fisher Scientific (A21208)	1:500
Donkey ECL-**anti-mouse HRP**	GE Healthcare (NA931V)	1:5000
Donkey ECL-**anti-rabbit HRP**	GE Healthcare (NA934V)	1:5000

ADDITIONAL REFERENCES

1. Cermak, T.; Doyle, E.L.; Christian, M.; Wang, L.; Zhang, Y.; Schmidt, C.; Baller, J.A.; Somia, N.V.; Bogdanove, A.J.; Voytas, D.F. Efficient design and assembly of custom TALEN and other TAL effector-based constructs for DNA targeting. *Nucleic Acids Res.* **2011**, *39*, e82.

2. Higgins, G.M.; Anderson, R.M. Experimental pathology of the liver. I. Restoration of the liver of the white rat following partial surgical removal. *Arch. Pathol.* **1931**, *12*, 186–202.
